# PYCR1 and PYCR2 Interact and Collaborate with RRM2B to Protect Cells from Overt Oxidative Stress

**DOI:** 10.1038/srep18846

**Published:** 2016-01-06

**Authors:** Mei-Ling Kuo, Mabel Bin-Er Lee, Michelle Tang, Willem den Besten, Shuya Hu, Michael J. Sweredoski, Sonja Hess, Chih-Ming Chou, Chun A. Changou, Mingming Su, Wei Jia, Leila Su, Yun Yen

**Affiliations:** 1 Department of Molecular Pharmacology, Beckman Research Institute at City of Hope, Duarte, CA 91010, USA; 2 Division of Biology and Biological Engineering, California Institute of Technology, Pasadena, CA 91125, USA; 3 Proteome Exploration Laboratory, Division of Biology and Biological Engineering, Beckman Institute, California Institute of Technology, Pasadena, CA, 91125; 4 Department of Biochemistry and Molecular Cell Biology, School of Medicine, College of Medicine, Taipei Medical University, Taipei, Taiwan 110; 5 Integrated Laboratory, Center of Translational Medicine, Taipei Medical University, Taipei, Taiwan 110; 6 Graduate Institute of Translational Medicine, College of Medical Science and Technology, Taipei Medical University, Taipei, Taiwan 110; 7 Graduate Institute of Cancer Biology and Drug Discovery, College of Medical Science and Technology, Taipei Medical University, Taipei, Taiwan 110; 8 University of Hawaii Cancer Center, HI 96813, USA

## Abstract

Ribonucleotide reductase small subunit B (RRM2B) is a stress response protein that protects normal human fibroblasts from oxidative stress. However, the underlying mechanism that governs this function is not entirely understood. To identify factors that interact with RRM2B and mediate anti-oxidation function, large-scale purification of human Flag-tagged RRM2B complexes was performed. Pyrroline-5-carboxylate reductase 1 and 2 (PYCR1, PYCR2) were identified by mass spectrometry analysis as components of RRM2B complexes. Silencing of both PYCR1 and PYCR2 by expressing short hairpin RNAs induced defects in cell proliferation, partial fragmentation of the mitochondrial network, and hypersensitivity to oxidative stress in hTERT-immortalized human foreskin fibroblasts (HFF-hTERT). Moderate overexpression of RRM2B, comparable to stress-induced level, protected cells from oxidative stress. Silencing of both PYCR1 and PYCR2 completely abolished anti-oxidation activity of RRM2B, demonstrating a functional collaboration of these metabolic enzymes in response to oxidative stress.

Ribonucleotide reductase (RR) catalyzes conversion of ribonucleoside diphosphate (NDP) to deoxyribonucleoside diphosphate (dNDP), a rate-limiting step in *de novo* synthesis of deoxyribunucleotide (dNTP). Hence, RR activity is crucial for maintaining cellular levels of dNTPs, which are used for DNA synthesis during DNA replication and DNA repair. Mammalian RR holoenzyme consists of two large subunits, RRM1, and two small subunits, RRM2 or RRM2B. In most cell types, expression of RRM1 is constant throughout all phases of cell cycle, whereas RRM2 expression is low in G1 phase^1^,^2^, induced during G1/S transition, and degraded in G_2_/M^3^ and in G_1_^4^ phase of the next cell cycle. RRM2B, also known as p53R2, was first identified as a p53-response gene^5^,^6^. Although both RRM2 and RRM2B are highly homologous, regulation of RRM2B is distinct from that of RRM2. Basal expression of RRM2B is low under unstressed condition, but is profoundly induced by stress such as DNA damage or oxidative stress^5^,^6^,^7^. Consistent with the modes of regulation, RRM1/RRM2 complex controls progression from G_1_ to S phase, whereas RRM1/RRM2B complex regulates DNA repair^8^,^9^,^10^.

Although it is well characterized that RRM2B is involved in the process of DNA repair, mutations of RRM2B have been identified in patients with mitochondrial DNA depletion syndrome, suggesting that RRM2B is an essential gene for the maintenance of mitochondrial DNA^11^,^12^,^13^,^14^,^15^. RRM2B is expressed at low level independent of p53 activation and without expression of RRM2 in non-proliferating cells, RRM1/RRM2B complex is the only remaining RR holoenzyme that maintains dNTP pools to supply for mitochondrial DNA synthesis as well as DNA repair^16^. Along the same line, MEFs derived from *Rrm2b* null mice show attenuated dNTP pools following oxidative stress and depletion of mitochondrial DNA content^11^.

We previously reported that purified recombinant RRM2B protein alone has intrinsic catalase activity to convert hydrogen peroxide to water and oxygen in an *in vitro* biochemical assay^17^. Over-expression of RRM2B in cancer cells reduced intracellular reactive oxygen species (ROS) and protected the mitochondrial membrane potential against hydrogen peroxide, demonstrating RRM2B’s involvement in anti-oxidation^17^. Consistent with this observation, silencing RRM2B in primary human fibroblasts, IMR90 cells, caused an increase in ROS level, induction of p38MAPK/p53 stress response pathway, and premature senescence^7^. Although our data demonstrate that RRM2B protects cells from overt oxidative stress, the underlying mechanism that governs such function is not entirely understood. It is unclear whether RRM1/RRM2B associated RR activity is required to antagonize oxidative stress in the cells. Cancer cells and primary cells express RRM2, which theoretically should be able to maintain dNTP pools when RRM2B is silenced. Therefore, it is conceivable that the anti-oxidation function of RRM2B is not dependent on RRM1-associated catalytic activity to produce dNDP and instead, depends on the interaction with other cellular factors or RRM2B itself.

In this study, we employed purification of RRM2B complexes followed by mass spectrometry analysis to identify novel RRM2B-associating factors, which might collaborate with RRM2B to antagonize overt oxidative stress. Using this approach, we successfully identified Pyrroline-5-carboxylate reductase 1 and 2 (PYCR1, PYCR2) as RRM2B-interactors. Most importantly, we formally demonstrated functional cooperation, either directly or indirectly, between RRM2B, PYCR1 and PYCR2 in response to oxidative stress.

## Results

### Purification of Human RRM2B Complexes

**Figure 1:**
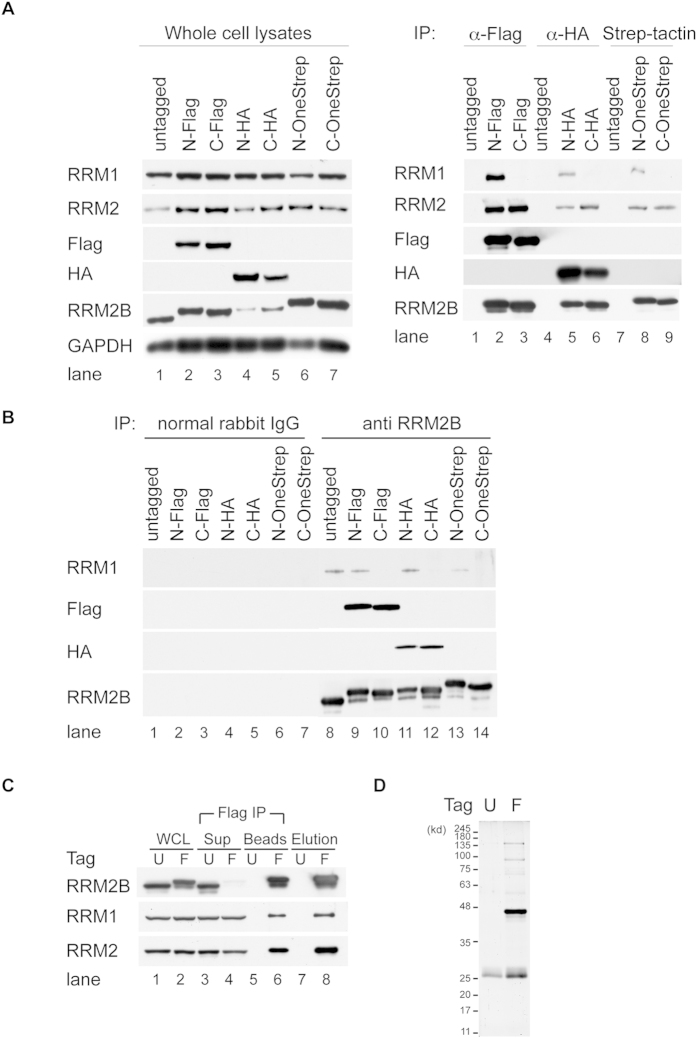
Comparison of Various Epitope-tagged RRM2B Proteins and Purification of Flag-tagged RRM2B Associated Complexes from 293 T-REx Cells. (**A**) Expression of untagged or epitope-tagged RRM2B proteins in 293 T-REx cells was induced with doxycycline (DOX) for 24 hours. Whole cell lysates (WCL) were precipitated with antibodies to Flag or HA beads as well as Strep-Tactin resins. (**B**) Same cell lysates in (**A**) were precipitated with RRM2B antibodies or control rabbit IgG. N-: epitope-tagged at the N-terminus; C-: epitope-tagged at the C-terminus (**C**) Large-scale immunoprecipitation (IP) was performed using whole cell lysates (WCL) from 293 T-REx-Flag-RRM2B cells (**F**) or control cells expressing untagged RRM2B (U) treated with DOX for 24 hours. A small fraction from each step during purification was saved for analysis. Sup: supernatant following IP; Beads: complexes pulled-down by Flag beads before elution and Elution: urea-eluted complexes. Denatured whole cell lysates and immune complexes electrophoretically separated on gels were transferred to membranes and blotted with antibodies to Flag, HA and RR subunits. (**D**) A small fraction of protein complexes after urea-elution was separated on a gel by electrophoresis, stained with SYPRO Ruby, visualized using UV-transilluminator and photographed by CCD camera.

To purify RRM2B complexes and identify associating factors, we first established several stable human 293 T-REx cell lines expressing RRM2B proteins, which were either untagged or tagged with Flag-, hemagglutinin (HA)- or OneStrep-epitope at the N- or C-terminus, upon induction by doxycycline (see Supplementary Fig. S1). RRM2B expression levels among all cell lines were equivalent. Immunoprecipitation (IP) by anti-Flag-, anti-HA- or StrepTactin beads followed by Western blotting was performed to compare the efficiency of pull-down. Flag antibody immunoprecipitated equal amounts of N-Flag- and C-Flag-tagged RRM2B. However, Flag-tag at the C-terminus of RRM2B greatly reduced co-IP of RRM1 without compromising association with RRM2 (Fig. 1A, right panel, lane 1 and 2). Similarly, RRM1 remained associated with RRM2B proteins that were tagged with HA or OneStrep at the N-termini, but not with those tagged at the C-termini (Fig. 1A, right panel, lane 5, 6, 8 and 9). Consistently, RRM2 remained associated with RRM2B proteins tagged with HA or OneStrep at the C-termini. IP by RRM2B antibody confirmed interference of RRM1 association by epitope tagging at the C-terminus of RRM2B (Fig. 1B, lane 8–14). It is likely that presence of additional amino acids at the C-terminus mask the adjacent RRM1-association domain^18^.

Since anti-Flag-beads immunoprecipitated RRM2B complex most efficiently, compared to anti-HA and StrepTactin beads and N-terminal tagging preserved complex formation with RRM1, a large-scale purification of RRM2B complexes was performed from whole cell lysate of 293 T-REx-Flag-RRM2B cells. 293 T-REx cells expressing untagged RRM2B were used as negative control. Flag-RRM2B complexes were first immunoprecipitated by anti-Flag-beads and eluted with urea. An aliquot from each step was examined by Western blotting to validate the purification procedure (Fig. 1C). Flag-RRM2B, RRM1 and RRM2 were specifically pulled down by Flag antibodies (Fig. 1C, lane 6) and eluted with urea (Fig. 1C, lane 8). Purified Flag-RRM2B associated complexes were separated eletrophoretically on SDS-PAGE and visualized by Sypro-Ruby staining. Several specific RRM2B-associated protein bands could be detected on the gel (Fig. 1D).

### Identification of RRM2B-associated Proteins by Mass Spectrometry

A mixture of urea-eluted RRM2B complexes was subjected to protease digestion and analyzed by mass spectrometry. Data analysis identified 154 candidate proteins. The list of the candidate proteins was then sorted by the intensity ratio of the Flag-RRM2B sample and the negative control. Among 154 candidates, 96 proteins had an intensity ratio of more than 10. The top three hits were the bait, RRM2B, and known associating protein RRM1 and RRM2, thus demonstrating that our methodology was able to pull down *bona fide* RRM2B associating-factors. Other than RR subunits, we did not identify those RRM2B- or RR-associated proteins reported previously from our group or others using different approaches^3^,^19^,^20^,^21^,^22^,^23^,^24^,^25^. Pyrroline-5-carboxylate reductase 1 and 2 (PYCR1, PYCR2) were among the top 10 candidates. PYCR1 and PYCR2 isozymes catalyze NAD(P)H-dependent conversion of Δ^1^-pyrroline-5-carboxylate (P5C) to proline. Given that PYCR1 and PYCR2 as well as proline are known to mediate anti-oxidation activity *in vivo*^26^,^27^, we focused on the biochemical and functional interactions of PYCR1 and PYCR2 with RRM2B.

**Figure 2:**
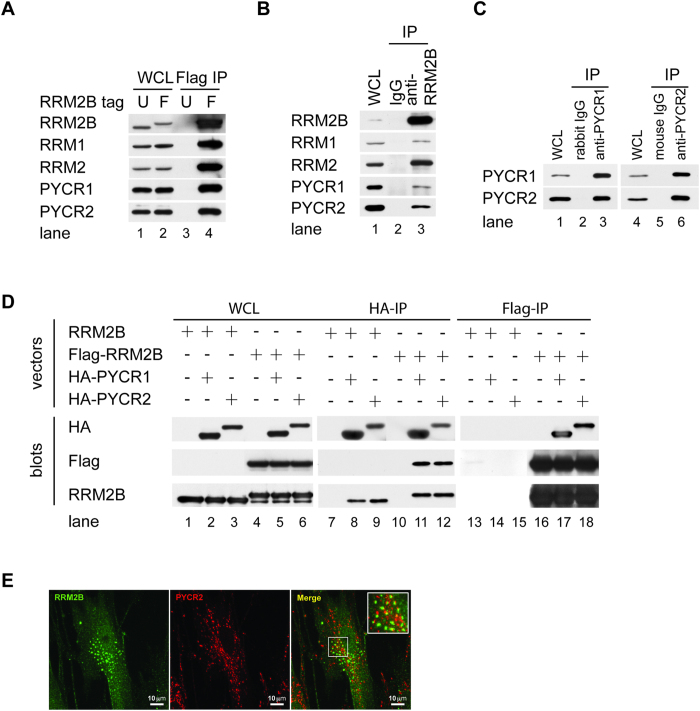
PYCR1 and PYCR2 form Complexes with RRM2B. (**A**) 293T REx-RRM2B or-Flag-RRM2B cells were treated with DOX and lysed after 24 hours. WCL was precipitated with Flag antibody. (**B**) Proliferating 293T cells were lysed and WCL was immunoprecipiated with antibodies to RRM2B or control rabbit IgG (**C**) WCL from 293T was immunoprecipitated with antibodies to PYCR1, PYCR2 or control rabbit or mouse IgG. (**D**) 293T cells were co-transfected with either RRM2B or Flag-RRM2B vectors and HA-PYCR1, or HA-PYCR2 vectors and lysed 48 hours later. Whole cell lysates were subjected to immunoprecipitation with antibodies to HA or Flag. Denatured whole cell lysates and immune complexes electrophoretically separated on gels were transferred to membranes and blotted with antibodies to RR subunits, PYCR1, PYC2, HA and Flag. (**E**) HFF-hTERT cells transduced with retroviruses expressing Flag-RRM2B were fixed and analyzed by indirect immunofluorescence. RRM2B (far red and pseudo-colored with green; left panel) and PYCR2 (red; middle panel) were visualized by confocal microscopy. Co-localization of RRM2B and PYCR2 was observed in the merged image (right panel).

We first validated the biochemical interactions of RRM2B with PYCR isozymes by IP-Western using various systems. Flag-IP from 293 T-REx-Flag-RRM2B cells pulled down all RR subunits as well as PYCR1 and PYCR2, validating our mass spectrometry data (Fig. 2A, lane 4). To exclude that any interaction was due to an artifact of RRM2B overexpression, we performed RRM2B-IP to pull down endogenous RRM2B complex from 293T cells. Endogenous PYCR1 and PYCR2 as well as RRM1 and RRM2 were co-immunoprecipitated with RRM2B antibodies (Fig. 2B, lane 3). However, reciprocal IP using highly specific PYCR1 and PYCR2 antibody did not pull down RRM2B (data not shown), It is likely that only an extremely small fraction of PYCR1 or PYCR2 was bound to RRM2B and IP by these antibodies was not efficient enough to detect complexes at such low level. Interestingly, we found that PYCR1 and PYCR2 were reciprocally co-immunoprecipitated, implicating that these two isozymes might form a complex *in vivo* (Fig. 2C). Lastly, we transiently co-expressed HA-tagged PYCR1 or PYCR2 with either untagged RRM2B or Flag-tagged RRM2B in 293T cells. Flag-tagged RRM2B was specifically co-immunoprecipitated by HA antibody, and HA-tagged PYCR proteins were co-immunoprecipitated by Flag antibody (Fig. 2D). Our biochemical approach clearly demonstrated interactions of PYCR1 and PYCR2 with RRM2B *in vivo*.

In addition to PYCR1 and PYCR2, pyrroline-5-carboxylate reductase-like (PYCRL) is the third member of PYCR protein family. PYCR1 and PYCR2 are closely related (85% identity), whereas PYCRL is the most distant member (it shares 45% homology with PYCR1 and PYCR2). Unlike PYCR1 and PYCR2 which are located in mitochondria, PYCRL is a cytosolic protein and utilizes cytosolic pool of P5C to produce proline^28^. We did not identify PYCRL from mass spectrometry analysis and confirmed that PYCRL was expressed at undetectable levels by Western blotting. To test whether there was specific interaction between PYCR members with RRM2B, we transiently co-expressed HA-tagged PYCRL with untagged or Flag-tagged RRM2B in 293T cells and tested the interaction by IP-Western. Similar to PYCR1 and PYCR2, we found that PYCRL, when overexpressed was able to form complex with RRM2B (see Supplementary Fig. S2). Supporting our biochemical data, PYCR1 and RRM2B complex formation was predicted by computational modeling analysis. The best docking model shows that RRM2B fills in to contact PYCR1 pentamer chains through their intermolecular binding domains (see Supplementary Fig. S3).

Finally, we performed indirect immunofluorescent staining followed by confocal microscopy to analyze cellular localization of both RRM2B and PYCR2 in HFF-hTERT cells. We observed distinct RRM2B-punctate staining in the cytoplasm and faint staining throughout the cells (left panel, Fig. 2E). PYCR2-punctate staining was observed in the cytoplasm only (middle panel, Fig. 2E). Partial colocalization of RRM2B and PYCR2 was evident in the merged image (right panel, Fig. 2E) supporting our biochemical data.

### Colocalization of RRM2B with PYCR1 and PYCR2 in Mitochondria

**Figure 3:**
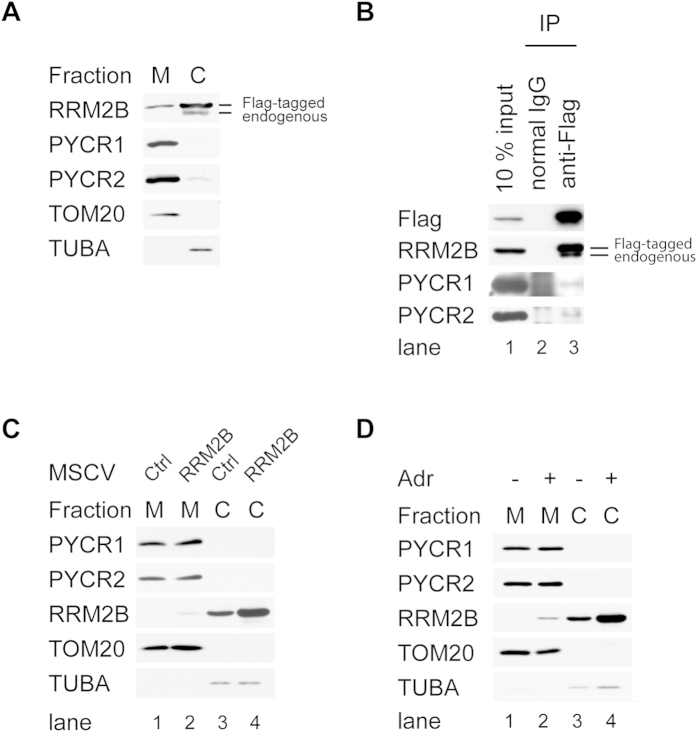
Co-localization of RRM2B with PYCR1 and PYCR2 in Mitochondria. (**A**,**B**) Mitochondrial fraction (M) and cytosolic (C) fraction were isolated from 293 T-REx-Flag-RRM2B cells, (**C**) HFF-hTERT cells transduced with MSCV retroviruses expressing either empty control or RRM2B and (**D**) HFF-hTERT cells treated with Adriamycin (Adr) (+) or left untreated (−) for 24 hours. (**A**, **C**, and **D**) Equal amount of proteins from each fraction separated electrophoretically on gels was transferred to membranes and blotted with antibodies. TOM20 was used as mitochondrial marker and α-TUBULIN (TUBA) was used as cytosolic maker to validate purity of each fraction. (**B**) Proteins from mitochondrial fraction were subjected to immunoprecipitation with normal mouse IgG or anti-Flag antibodies. Western blot analysis was performed to detect Flag-RRM2B, RRM2B, PYCR1 and PYCR2.

Although RRM2B has been implicated in a group of mitochondrial DNA depletion syndromes^11^,^12^,^13^,^14^,^15^,^29^, it has not been formally demonstrated that this protein is located in the mitochondria. To demonstrate co-localization of PYCR proteins with RRM2B in mitochondria, we performed cellular fractionation from 293 T-REx-Flag-RRM2B cells. Proteins in the cytosolic and mitochondrial fractions were detected by Western blot analysis. The majority of PYCR1 and PYCR2 were detected in the mitochondria as reported by others^28^. In contrast, both Flag-RRM2B and endogenous RRM2B were primarily located in the cytosol. However, a fraction of RRM2B was readily detected in the mitochondrial compartment (Fig. 3A). Immunoprecipitation of mitochondrial proteins by RRM2B antibody pulled down both PYCR1 and PYCR2, indicating that interaction likely takes place in mitochondria (Fig. 3B).

We further investigated cellular localization of RRM2B, PYCR1 and PYCR2 in hTERT-immortalized normal human foreskin fibroblast cell line (HFF-hTERT) in which the stress response pathway remains intact. Very low amount of RRM2B was detected in the cytosolic fraction in the unstressed cells and undetectable in the mitochondrial fraction. When overexpressed, small fraction of RRM2B was detected in the mitochondria (Fig. 3C). To investigate whether RRM2B could be located in the mitochondria under any physiological relevant condition, we treated HFF-hTERT cells with a DNA damaging agent, Adriamycin, to elevate expression of endogenous RRM2B. Unlike RRM2B, PYCR1 and PYCR2 expression levels and cellular localization were unchanged following Adriamycin treatment (Fig. 3D) in HFF-hTERT cells. After Adriamycin treatment, a moderate level of RRM2B was detected in the mitochondrial fraction where both PYCR1 and PYCR2 were located, implicating RRM2B activity in mitochondria under stress conditions.

### PYCR Isozymes are Essential for Cell Proliferation in Normal Human Fibroblast Cell Line and Embryonic Development in Zebrafish

**Figure 4:**
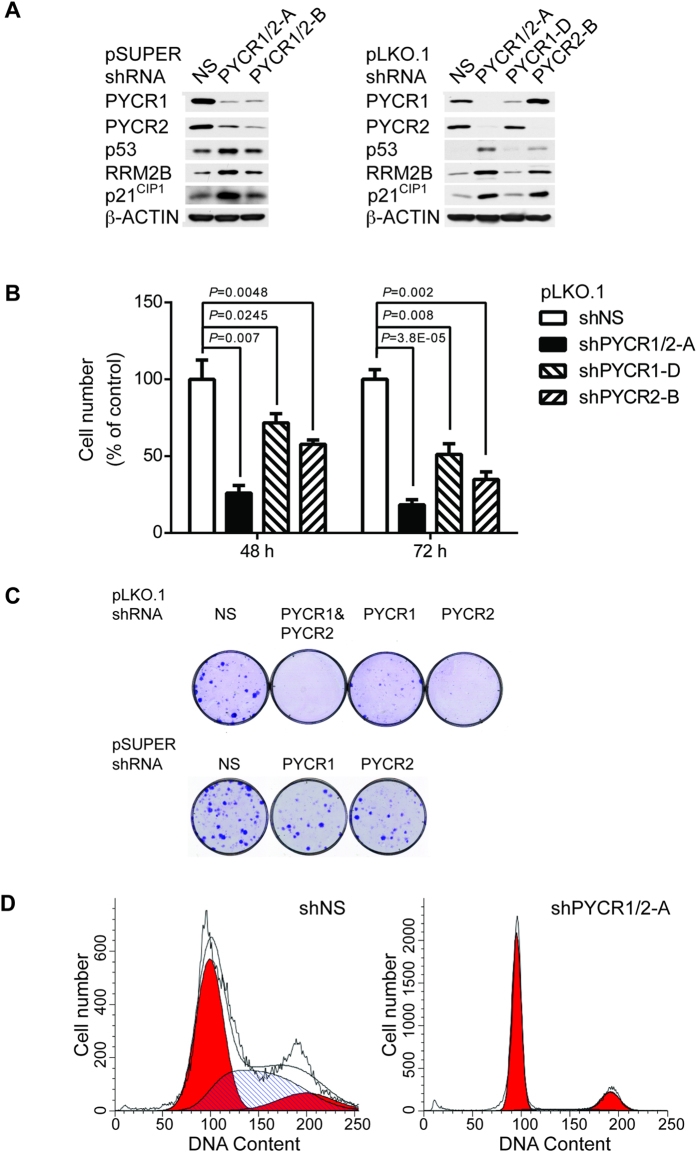
Activation of p53 Pathway and Cell Cycle Arrest by Silencing of PYCR1 and PYCR2. (**A**) HFF-hTERT cells were transduced with pSUPER.retro.puro retroviruses (left panel) expressing shRNAs that silenced both PYCR1 and PYCR2 or with pLKO.1 lentiviruses (right panel) expressing shRNAs that silenced PYCR1 or PYCR2 alone or both. Non-silencing (NS) shRNA was used as control for each vector. Four days later, cell lysates were immunoblotted with antibodies to PYCR1, PYCR2, p53, RRM2B and p21^CIP1^. (**B**) HFF-hTERT cells transduced with pLKO.1 lentiviruses were seeded in 6-well plates (n = 3) four days after infection. Cell numbers were scored 48 and 72 hours later. Data were plotted as % of shNS control for each time point. (**C**) HFF-hTERT cells transduced with either pLKO.1 (top panel) or pSUPER.retro.puro (bottom panel) were seeded at low density in 60 mm-dishes four days after infection. Colonies were fixed and stained 2 weeks later. (**D**) HFF-hTERT cells transduced with pLKO.1 lentiviruses expressing shNS (left panel) or shPYCR1/2-A (right panel) were stained with PI solution and analyzed by FACS 6 days post-infection.

Both PYCR1 and PYCR2 are involved in proline synthesis. Hence, it is conceivable that deficiency of these enzymes can trigger stress response in normal human cells. To test the hypothesis, we expressed multiple shRNAs by pSUPER.retro.puro retroviral or pLKO.1 lentiviral vectors to silence expression of PYCR1 or PYCR2 in HFF-hTERT cells (see Supplementary Fig. S4). While some of these vectors were able to silence specific PYCR genes, some of the shRNAs indeed targeted both PYCR1 and PYCR2 (Fig. 4A and see Supplementary Fig. S4), possibly due to high homology of RNA sequences (see Supplementary Fig. S4). We found that silencing of PYCR1 or PYCR2 impeded cell proliferation, and co-silencing of PYCR1 and PYCR2 triggered robust proliferation defect within one week (Fig. 4B). Accordingly, long-term silencing of both PYCR1 and PYCR2 dramatically reduced the colony formation, compared to expression of non-silencing control, shNS by both vectors. In contrast, silencing of either PYCR1 or PYCR2 displayed intermediate phenotype (Fig. 4C). Cell cycle analysis showed that silencing of both PYCR1 and PYCR2 caused profound reduction of cells in S phase and the majority of cells were arrested in G1 phase, and a small fraction of cells were in G2/M phase compared to shNS control (Fig. 4D).

P53 tumor suppressor senses stress signals and activates a downstream transcription program to trigger various biological responses to cope with stress such as cell cycle arrest, DNA repair, apoptosis, autophagy, senescence and metabolism^30^,^31^. We found that p53 protein level was significantly increased in cells expressing shRNAs that silenced both PYCR1 and PYCR2 (Fig. 4A). Downstream targets of p53 such as p21^CIP1^ and RRM2B were also induced, suggesting activation of p53 pathway (Fig. 4A). Similarly, silencing of PYCR2 alone was able to activate p53 pathway. However, we were unable to detect significant elevated levels of p53, p21^CIP1^ or RRM2B in shPYCR1-expressing cells. It remains to be investigated whether this is due to incomplete silencing of PYCR1, or PYCR1 and PYCR2 having specific functions.

To further investigate the role of all three PYCR isozymes *in vivo*, we knocked down *pycr* gene expression in zebra fish by morpholino oligonucleotides (MO) targeted against the 5′-untranslated region of the mRNA individually or in combination. Co-injection of two *pycr* MO caused profound defects during embryonic development demonstrating the essential functions of *pycr* isozymes *in vivo* (see Supplementary Fig. S5 and S6).

### Silencing of PYCR1 and PYCR2 Sensitized HFF-hTERT Cells to Hydrogen Peroxide

**Figure 5:**
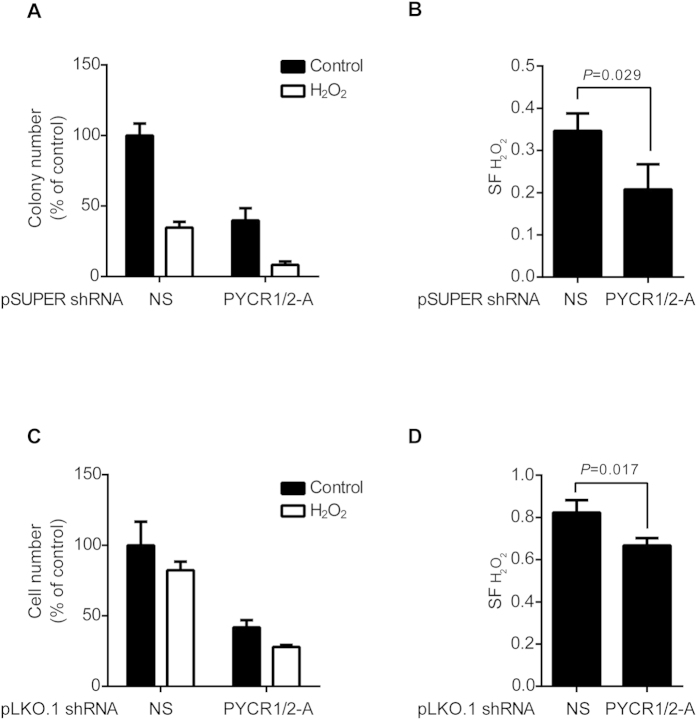
Silencing of PYCR1 and PYCR2 Induced Hypersensitivity to H_2_O_2_ in HFF-hTERT Cells. (**A**) HFF-hTERT cells were transduced with pSUPER.retro.puro retroviruses expressing shRNAs silencing both PYCR1 and PYCR2 or non-silencing (NS) control. Cells were seeded at the low density four days post-infection and treated with 200 μM H_2_O_2_ for 10 minutes next day. Colonies were stained and total colony numbers were scored after two weeks. Data were plotted as % of shNS control or (**B**) surviving fraction (SF) after treatment of 200 μM H_2_O_2_. (n = 3) (**C**) HFF-hTERT cells were transduced with pLKO.1 lentiviruses expressing shRNAs silencing both PYCR1 and PYCR2 or non-silencing (NS) control. Cells of equal number were seeded at the low density four days post-infection. Cells were treated with 50 μM H_2_O_2_ for 10 minutes the next day and cell numbers were scored after two days. Data were plotted as % of shNS control or (**B**) surviving fraction (SF) after treatment of 50 μM H_2_O_2_. (n = 3).

Fibroblasts deficient in PYCR1, derived from patients with cutis laxa, showed increased sensitivity to oxidative stress. Heightened sensitivity was characterized by increased cell death, changes in mitochondrial morphology, and reduced mitochondrial membrane potential following treatment of hydrogen peroxide^27^. To test whether specific silencing of both PYCR1 and PYCR2 had any impact on sensitivity to overt oxidative stress in normal cells, we performed colony formation assay on HFF-hTERT cells transduced with pSUPER.retro.puro retroviruses that express shRNA and silence both PYCR1 and PYCR2 or shNS control. We found that reduced expression of PYCR1 and PYCR2 caused sensitization of cells to hydrogen peroxide at concentrations where very low or no Fenton reaction occurs (200 and 50 μM, respectively, in Fig. 5A,B). To further confirm our observation, we tested HFF-hTERT cells expressing shPYCR1/2 by pLKO.1 vector, using a short-term proliferation assay. Accordingly, HFF-hTERT cells devoid of any expression of PYCR1 and PYCR2 were more sensitive to overt oxidative stress than those expressing shNS (Fig. 5C,D). Our data demonstrated that PYCR1 and PYCR2 expression protect cells from overt oxidative stress.

### Silencing of Both PYCR1 and PYCR2 Caused Partial Fragmentation of Mitochondria

**Figure 6:**
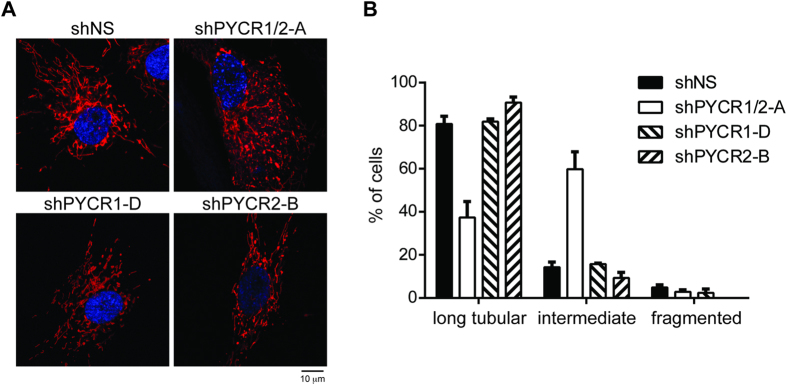
Silencing of Both PYCR1 and PYCR2 Caused Partial Fragmentation of Mitochondrial Network. (**A**) HFF-hTERT cells were first transduced with retrovirus expressing cox4-DsRed that labels mitochondria *in vivo*. DsRed positive cells were sorted and re-infected with pLKO.1 lentiviruses expressing shNS, shPYCR1/2-A, shPYCR1-D or shPYCR2-B. Mitochondria morphology was visualized by confocol microscopy. Representative photographs are presented. (**B**) At least 100 cells from each slide were examined and mitochondrial morphology was categorized in one of the three groups. (n = 3).

PYCR1 and PYCR2 are both located in mitochondria, where the bulk of metabolic reactions take place to support cellular functions and generate reactive oxygen species. PYCR1 also protects cells from mitochondrial fragmentation upon oxidative stress^27^. We examined the mitochondrial morphology of HFF-hTERT cells expressing shPYCR1, shPYCR2, shPYCR1/2 or shNS and determined whether the absence of both PYCR1 and PYCR2 had any impact on mitochondria structure. Mitochondrial fluorescent protein, cox4-DsRed, was expressed in HFF-hTERT cells to visualize mitochondrial structure *in vivo*. Mitochondrial morphology in shNS-, shPYCR1-, or shPYCR2-expressing HFF-hTERT cells was mostly long and tubular (Fig. 6A,B). However, the mitochondrial morphology in cells lacking PYCR1 and PYCR2 was partially fragmented, although long tubular structures remained present in the cells (Fig. 6A,B). Our data demonstrated that PYCR1 and PYCR2 play a role in maintaining mitochondrial structure under unstressed condition.

### Modulation of Antioxidative Function of RRM2B by PYCR1 and PYCR2

**Figure 7:**
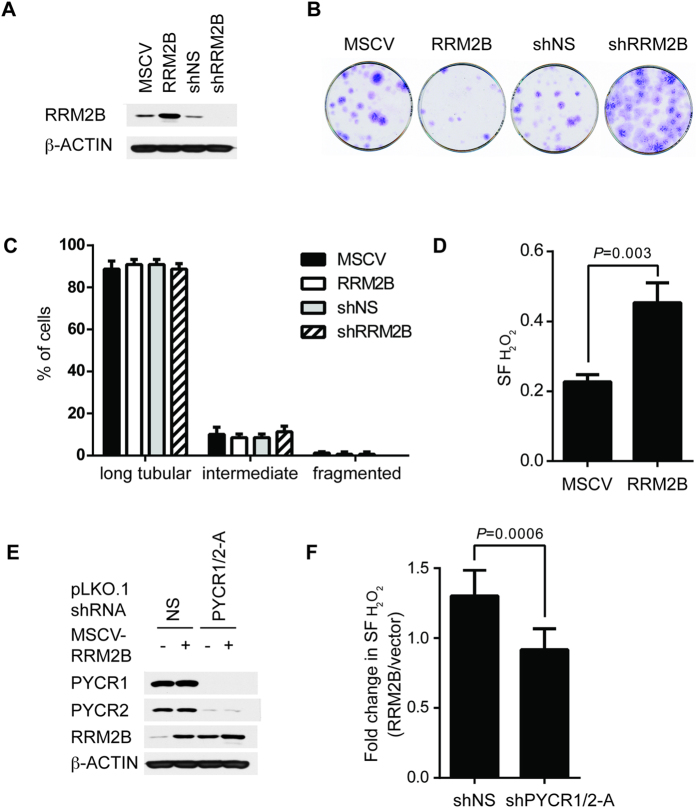
RRM2B Cooperates with PYCR1 and PYCR2 to Confer Resistance to H_2_O_2_ in HFF-hTERT Cells. (**A**) HFF-hTERT cells were transduced with retroviruses overexpressing or silencing RRM2B. Empty MSCV vector or pSUPER.retro.puro vector expressing non-silencing shRNA (shNS) were included as controls. Whole cell lysates were subjected to Western blot analysis to validate overexpression or silencing of RRM2B. β-ACTIN was used as control to ensure equal loading of each lane. (**B**) Transduced HFF-hTERT cells were seeded at low density. Colonies were stained two weeks later. (**C**) HFF-hTERT-cox4-DsRed cells were infected with viruses to overexpress (MSCV) or silence (pSUPER.retro.puro) RRM2B cells. Mitochondrial morphology was examined and scored as described in Figure 6. (**D**) HFF-hTERT cells overexpressing RRM2B or empty vector control were treated with 200 μM H_2_O_2_ for 10 minutes and the cell numbers were scored two days later. Data were plotted as surviving fraction after treatment of 200 μM H_2_O_2_. (n = 3) (**E**) HFF-hTERT cells were co-infected with MSCV and pLKO.1 viruses to overexpress RRM2B or silence PYCR1 and PYCR2. Whole cell lysates were subjected to Western blot analysis to validate overexpression or silencing. β-ACTIN Actin was used as control to ensure equal loading of each lane. (**F**) Transduced cells as described in (**E**) were treated with 200 μM H_2_O_2_ for 10 minutes and the cell numbers were scored two days later. Data were plotted as fold change in surviving fraction after treatment of 200 μM H_2_O_2_. (n = 6).

Although RRM2B was initially identified as a small RR subunit induced by DNA damage to supply dNTPs for DNA repair and mitochondrial DNA synthesis^5^,^6^,^16^,^32^, several lines of evidence suggest that RRM2B also has anti-oxidative stress functions^7^,^17^. Currently, the mechanism underlying such function remains unclear. Like RRM2B, proline and expression of functional PYCR1 and PYCR2 are involved in anti-oxidation in human fibroblasts and immortalized cell lines^26^,^27^. It is likely that RRM2B, PYCR1 and PYCR2 are functionally linked to anti-oxidative functions. To test this hypothesis, we silenced or overexpressed RRM2B in HFF-hTERT cells (Fig. 7A). RRM2B expression by pMSCVhyg vector was comparable to levels in Adriamycin-treated cells to recapitulate physiologically relevant conditions (Fig. 3D). Consistent with our previous observation in cancer cell lines^33^, overexpression of RRM2B in HFF-hTERT cells impeded cell proliferation, whereas silencing of low basal level of RRM2B with enhanced proliferation potential increased colony numbers (Fig. 7B). Since basal expression of RRM2B does not interact with PYCR1 and PYCR2, the growth inhibitory effect of low level of RRM2B is likely independent of PYCR isozymes. We next examined the mitochondrial morphology and found no impact of overexpression or silencing of RRM2B on the mitochondrial structure (Fig. 7C). Lastly, we tested sensitivity to hydrogen peroxide by short-term proliferation assay and found that HFF-hTERT cells expressing higher level of RRM2B were more resistant to oxidative stress (Fig. 7D). To test whether anti-oxidation function of RRM2B was dependent on functional PYCR1 and PYCR2, we expressed an empty vector, RRM2B in combination with shNS or shPYCR1/2-A in HFF-hTERT cells (Fig. 7E). RRM2B overexpressing cells were no longer resistant to hydrogen peroxide, when both PYCR1 and PYCR2 were silenced in the cells (Fig. 7F), thus demonstrating that anti-oxidation function of RRM2B requires the presence of PYCR1 and PYCR2. Consistent with this conclusion, PYCR1 and PYCR2-deficient cells were more sensitive to hydrogen peroxide despite elevated level of RRM2B (Fig. 4A and Fig. 7E). It remains to be further investigated whether PYCR1 and PYCR2 directly mediate RRM2B’s activity in antagonizing oxidative stress or indirectly through downstream events.

## Discussion

RRM2B forms complexes with RRM1 to constitute RR activity in the *de novo* synthesis pathway of dNTPs for DNA repair or mitochondrial DNA synthesis upon stress stimulation and during the quiescence state, respectively^16^. Several lines of evidence suggest that high levels of RRM2B reduce reactive oxygen species and maintain mitochondrial membrane potential under normal physiological or oxidative environment^7^,^17^. It is highly plausible that prevention of overt oxidative states in cells by RRM2B is due to its role in DNA repair and maintenance of mitochondrial DNA content. Positive feedback regulation exists between DNA damage and generation of ROS, by which induction of p53 from stress triggers downstream pro-oxidation genes^34^. Expression of high RRM2B levels could suppress induction of the feedback control by supplying sufficient dNTPs to reduce the level of DNA damage and protect mitochondria from high levels of ROS. Nevertheless, whether RRM2B mediates anti-oxidative function through proteins other than RRM1 in cells remains unclear. In this report, we employed a biochemical approach to identify novel associating factors that might link RRM2B to anti-oxidation.

As proof of principle, we identified known RRM2B-associating factor, RRM1, from our mass spectrometry analysis. Interestingly, RRM2 was also identified as part of RRM2B complexes. It is not clear whether RRM2-RRM2B complex is present with or without association with RRM1 in the cells and whether it is functional. Since RRM2B and RRM2 are highly homologous^5^,^6^ and have similar biochemical properties^8^,^35^,^36^, it is conceivable that RRM2 and RRM2B form a functional hetero-dimeric subunit of RR in promoting ribonucleotide reduction. Our IP experiments were performed using 293T cells in which both RRM2 and RRM2B are expressed at high level. In contrast, RRM2B is expressed at low level in proliferating normal human cell strain in which RRM2 expression is high^7^. Conversely, RRM2B is highly up-regulated under stress condition, whereas RRM2 expression declined due to cell cycle arrest^7^. Thus, the abundance and functional importance of RRM2-RRM2B hetero-complex might be cell system-dependent.

PYCR1 and PYCR2 were among the top candidates identified by mass spectrometry analysis of purified RRM2B. Interaction between RRM2B with PYCR1 and PYCR2, either endogenously or exogenously, was validated by IP-Western, using a variety of approaches and cells lines containing either functional or disabled p53. Although the majority of PYCR1 and PYCR2 are located in the mitochondria and most of RRM2B are cytosolic as reported by others^28^,^37^, we found that a small fraction of RRM2B is present and bound to PYCR1 and PYCR2 in the mitochondria when total RRM2B levels were elevated by either overexpression or induced by DNA damage. There are at least three RRM2B protein isoforms that differ in length and sequence at the N-terminus. Isoform 1 is the most characterized and was studied in this report. Although isoform 1 could be detected in the mitochondria, it contains no predicted mitochondria targeting sequence (MTS). It is likely that a non-typical MTS exists in isoform 1 or RRM2B-associated proteins and facilitates translocation into the mitochondria with low efficiency. Additionally, post-translational modification of RRM2B protein triggered by DNA damage response might enhance translocation of RRM2B from cytosol to mitochondria and interaction with PYCR1 and PYCR2. Interestingly, a predicted MTS was identified at the N-terminus of isoform 2, which may translocate into mitochondria more efficiently and have an essential function in mitochondria (see Supplementary Fig. S7).

Our loss-of-function studies showed that silencing of PYCR1 and PYCR2 in hTERT-immortalized human fibroblasts activated p53 pathway and caused a proliferation defect. P53 is a stress-sensor that can be activated by a variety of stress signals such as DNA damage, oxidative stress, oncogenic stress or nutrient deprivation^30^,^31^. The exact type of stress that activate p53-stress response pathway in PYCR1 and PYCR2 deficient HFF-hTERT cells remains to be elucidated. It has been reported that cancer cells reprogram proline and glutamine metabolism to promote proliferation^38^. Silencing of c-Myc proto-oncogene in Burkitt’s lymphoma cancer cells suppressed expression of PYCR1, which promoted synthesis of proline from P5C, and induction of proline oxidase (POX), P5C dehydrogenase (P5CDH) and glutamine synthetase (GS). This ultimately catalyzed conversion of proline to P5C, glutamate and glutamine in three sequential reactions^38^. Hence, c-Myc expressing cells exhibited higher level of proline^38^. Furthermore, silencing of PYCR1 by shRNA in breast cancer cells reduced tumor-forming capacity in a mouse model, demonstrating the role of PYCR in cancer cell growth *in vivo*^39^. It is unclear whether an increased level of proline is the sole requirement to fuel cancer cell growth. But, it seems unlikely that simply promoting conversion from one non-essential amino acid to another promotes cell proliferation.

To establish correlation between cell cycle progression defect induced by silencing of PYCR1 and PYCR2 and cellular levels of proline or other amino acids, we performed targeted metabolomic analysis from HFF-hTERT cells expressing shNS or shPYCR1/2. We found that cells deficient in PYCR activity did not significantly reduce steady state proline level (see Supplementary Fig. S8). Interestingly, the level of 4-hydroxyproline was significantly reduced in shPYCR1/2 expressing cells. Free proline pool is relatively low in the cells and instead, most of proline synthesized is stored in the proline-rich extracellular matrix protein such as collagen. Once incorporated, proline is hydroxylated by post-translational modification. Depletion of 4-hydroxyproline might reflect an increase in conversion of 4-hydroxyproline to proline when *de novo* synthesis of proline was blocked. Our data demonstrated that a proline-independent mechanism might have contributed to cell cycle arrest-induced by PYCR1 and PYCR2 silencing. PYCR1 and PYCR2 use NADH as cofactor and generate NAD + while catalyzing synthesis of proline from P5C^28^. Therefore, it is likely that the essential role of PYCR1 and PYCR2 during cell proliferation is to channel NADH/NAD + to other core metabolic pathways such as glycolysis. Disruption of cell metabolism or redox homeostasis is likely to activate p53-induced downstream events such as apoptosis, senescence, or cell cycle arrest^40^,^41^.

Apart from their role in metabolism, PYCR1 and PYCR2 were found in our study as essential in maintaining integrity of normal mitochondrial structure. Proliferating HFF-hTERT cells displayed mostly long-tubular mitochondria morphology, whereas silencing of both PYCR1 and PYCR2 caused fragmentation of mitochondria, which might transduce stress signal to activate p53 and downstream events. It is unclear whether mitochondrial fragmentation in PYCR1 and PYCR2 deficient cells was due to induction of mitophagy or imbalance of mitochondrial fission and fusion dynamics. Consistent with our observation, fibroblasts derived from PYCR1 mutated patients have been characterized with increased fragmented mitochondrial structure, but only after exposure to hydrogen peroxide^27^. PYCR2 alone might be sufficient to maintain normal mitochondrial structure when PYCR1 is absent under physiological conditions and vise versa.

PYCR1 and PYCR2 are expressed constitutively at high levels in the mitochondria, whereas RRM2B is expressed at very low levels in the cytosol under normal physiological condition, thus suggesting that PYCR and RRM2B have independent functions without stimulation by stress signals. This is supported by our observation that, unlike PYCR1 and PYCR2, RRM2B is not involved in maintaining normal mitochondrial structure and does not promote cell proliferation without environmental stress. However, PYCR1, PYCR2 and RRM2B share common anti-oxidation activities such as suppressing level of ROS, maintaining mitochondrial potential and promoting cell survival in human cancer cells, immortalized normal human cells or primary human fibroblasts as reported in our current study and previously^7^,^17^,^26^,^27^. Furthermore, increased levels of oxidized forms of methionine, methionine sulfoxide, was observed in PYCR1 and PYCR2 as well as RRM2B silenced hTERT-HFF cells (see Supplementary Fig. S8), indicating that all of these enzymes control the balance of redox status in the cells. In this report, we demonstrated for the first time that the anti-oxidative function of RRM2B was abolished when both PYCR1 and PYCR2 were silenced, which establish a functional connection among these enzymes. Our data suggest that RRM2B works with PYCR isozymes to suppress oxidative stress-induced damage in normal cells either directly though protein-protein interaction or indirectly through downstream pathways. We previously reported that RRM2B has catalase activity, which detoxifies ROS^17^. Therefore, mitochondrial localization of RRM2B might play a role in protecting PYCR1 and PYCR2 proteins from ROS-induced damage and stabilizing functional catalytic activity under stress condition. Whether RRM2B and PYCR isozymes directly regulate each other’s activity remains to be investigated in the future using genetic and biochemical approaches. In particular, generations of RRM2B-mutants, which are unable to transport to mitochondria and interact with PYCR1 and PYCR2 or *vice versa*, are crucial to uncover the molecular mechanisms.

Taken together, our data demonstrated that PYCR1 and PYCR2 are essential for cell proliferation under normal physiological conditions, and stress-induced RRM2B interacts with PYCR1 and PYCR2 to antagonize oxidative insults and promote survival in normal cells. Furthermore, our loss-of-function study in zebrafish model provided the physiological importance of three *pycr* isozymes in embryonic development. It has been reported that PYCR1 was one of the most frequently overexpressed metabolic genes across 1,981 tumor samples spanning 19 cancer types, suggesting that cancer cells might be addicted to high level of PYCR1 to support cell growth and confer resistance to oxidative stress for cell survival^42^. Hence, PYCR1 is a potential target for novel chemotherapeutic agent development, not only as a means to inhibit tumor cell growth, but also to sensitize existing cancer cells that express RRM2B to ionizing radiation and other modalities.

## Methods

### Cell Culture and Chemicals

Human telomerase reverse transcriptase (hTERT)-immortalized normal human foreskin fibroblasts (HFF-hTERT) (a gift from Dr. Jeffrey S. Dome at Children’s National Medical Center, Washington, DC), Flp-In™ T-REx™ 293 cells and 293T cells were cultured in Dulbecco’s Modification of Eagle’s Medium (DMEM) (Mediatech, Inc., Manassas, VA, USA) supplemented with 10% fetal bovine serum (FBS) (Omega Scientific, Inc., Tarzana, CA, USA) and penicillin and streptomycin (Thermo Fisher Scientific Inc.). All cells were incubated at 37 °C and 5% CO_2_ in a humidified incubator. Adriamycin was obtained from the City of Hope Pharmacy (Duarte, CA, USA). Hydrogen peroxide was purchased from Avantor Performance Materials, Inc. (Center Valley, PA, USA). Doxycycline was purchased from Sigma-Aldrich (St. Louis, MO, USA).

### Plasmids

Untagged, Flag-, HA- and OneStrep-tagged human RRM2B cDNAs were generated by polymerase chain reaction (PCR) using oligonucleotides as primers listed in Supplementary Table S1. Two sequential steps of PCR were performed to generate OneStrep-tagged RRM2B at the N-terminus and C-terminus. PCR-amplified DNA fragments were digested with BamHI and XhoI and inserted into the same sites in pcDNA5/FRT/TO vector (Thermo Fisher Scientific Inc., Waltham, MA). Untagged RRM2B was inserted into BamHI and XhoI sties of the modified pMSCVhyg vector (Clontech Laboratories, Inc., Mountain View, CA, USA) in which NotI, BamHI, BstXI and SnaBI sites were inserted between BglII and XhoI sites. HA-tagged PYCR1, PYCR2 and PYCRL were generated by PCR. Primer sequences were listed in Supplementary Table 1. PCR-amplified DNA fragments were digested with BamHI and XhoI and inserted into the same sites in pcDNA3 (Thermo Fisher Scientific Inc., Waltham, MA, USA). All cDNA sequences were verified before transfection. Retroviral pSUPER.retro.puro vectors (OligoEngine, Seattle, WA, USA) and lentiviral pLKO.1 vectors (GE Healthcare Dharmacon Inc., Lafayette, CO, USA) were used to express shRNA. Target sequences were selected using siDESIGN Center siRNA design tool (dharmacon.gelifesciences.com/design-center) for pSUPER.retro.puro vector. Oligonucleotides were designed and inserted into pSUPER.retro.puro vector according to the instruction of the manufacture. pSUPER.retro.puro-shNS was described previously^7^. Lentiviral pLKO.1/TRC shPYCR1 and shPYCR2 vectors were purchased from GE Healthcare Dharmacon Inc.) Lentiviral pLKO.1-puro non-mammalian shRNA control plasmid was purchased from Sigma-Aldrich (St. Louis, MO). All target sequences are listed in Supplementary Table S2. Retroviral pclbw-cox4-Dsred vector expressing mitochondria-targeting red fluorescent protein from Discosoma sp. (DsRed) was a gift from Dr. David Chan at California Institute of Technology, Pasadena, CA.

### Generation of Stable Cell Lines Expressing Doxycycline-Inducible Untagged or Epitope Tagged-RRM2B Proteins

Flp-In™ T-REx™ 293 cells were co-transfected with pOG44 and pcDNA5/FRT/TO-untagged or -tagged RRM2B vectors. Transfected cells were selected with 100 μg/ml hygromycin for two weeks. Expression of RRM2B cDNA was induced by 1 μg/ml doxycycline for 24 hours.

### Retroviral and Lentiviral Production and Infection

Retroviruses and lentiviruses were generated as previously described^7^. HFF-hTERT cells were transduced with the VSV-G pseudo-typed retrovirus or lentivirus expressing cDNA or shRNAs and selected with 2 μg/ml puromycin (Sigma-Aldrich) or 100 μg/ml hygromycin (EMD Chemicals, Gibbstown, NJ, USA) prior to all assays.

### Large Scale Purification of Flag-tagged RRM2B Complexes and Digestion of Proteins

293 T-REx-RRM2B (negative control) and -Flag-RRM2B cells were incubated with 1 μg/ml doxycycline for 24 hours and harvested. Immunoprecipitation, elution and digestion of RRM2B protein complexes were carried out as described previously^43^.

### Mass Spectrometric and Data Analyses

Liquid chromatography-mass spectrometry and data analyses of the digested immunoprecipitated samples were carried out as previously described^44^ with modifications. Further details are reported in Supplemental Experimental Procedures.

### Subcellular Fractionation

Twenty million cells were harvested for sub-cellular fractionation. Dounce homogenization (80 strokes for HFF-hTERT cells and 110 strokes for 293 T-REx-RRM2B cells) and differential centrifugation were performed to separate mitochondria from cytosol components by using Mitochondria Isolation Kit for Cultured Cells (Thermo Fisher Scientific Inc.) and according to the instruction by the manufactor.

### Immunoprecipitation

Cells were rinsed with PBS and lysed with buffer A (50 mM HEPES, pH 7.5; 70 mM potassium acetate and 5 mM magnesium acetate) supplemented with protease inhibitor cocktail (Sigma-Aldrich) and phosphatase inhibitors (10 mM β-glycerophosphate, 1 mM sodium fluoride and 0.1 mM sodium vanadate) for 30 min on ice. Whole cell lysate was cleared by centrifugation for 10 min. Protein concentration was quantified by BCA kit (Thermo Fisher Scientific Inc.). Equal amount of total proteins was incubated with Strep-Tactin resins (IBA GmbH, Goettingen, Germany), ANTI-FLAG® M2 Affinity Gel (Sigma-Aldrich), anti-HA agarose (Sigma-Aldrich), normal mouse IgG (Santa Cruz Biotechnology Inc. Dallas, TX, USA), normal rabbit IgG (Santa Cruz Biotechnology Inc.) or antibodies to PYCR1 (Abgent, Inc., San Diego, CA), PYCR2 (LifeSpan BioSciences, Inc. Seattle, WA, USA), RRM2B (Rockland Immunochemicals Inc., Limerick, PA), for three hours to overnight at 4 °C with rocking. Protein A or protein G beads (Santa Cruz Biotechnology Inc.) were added to the antibody-antigen reaction for additional one hour. Beads were collected by centrifugation at 10,000x g for 15 seconds and then washed with ice-cold buffer A three times before boiling in SDS-PAGE sample buffer.

### Immunoblotting

Procedures for immunoblotting were as described^7^. Briefly, cells were lysed in RIPA buffer (150 mM sodium chloride, 1% NP-40, 0.5% sodium deoxycholate, 0.1% SDS and 50 mM Tris, pH 8.0), and protein was quantified by BCA assay (Thermo Fisher Scientific). Proteins separated on 10% polyacrylamide gel were transferred to PVDF membranes (Millipore, Billerica, MA) and detected using antibodies to PYCR1 (Abgent, Inc.), PYCR2 (LifeSpan BioSciences, Inc.), human RRM2B (Rockland Immunochemicals Inc.), RRM1 (T16; Santa Cruz Biotechnology Inc.), RRM2 (N-18; Santa Cruz Biotechnology Inc.), p21^CIP1^ (F-5; Santa Cruz Biotechnology Inc.), TOM20 (F-10; Santa Cruz Biotechnology Inc.), TUBA (clone YL1/2; Accurate Chemical & Scientific Corporation, Westbury, NY, USA), human p53 (EMD Chemicals, Gibbstown, NJ, USA) and β-ACTIN (Sigma-Aldrich).

### Immunofluorescence

HFF-hTERT cells transduced with retrovirus expressing Flag-RRM2B were seeded onto glass coverslips, fixed with 4% paraformaldehyde and permeabilized with 0.3% Triton X-100 in PBS. Cells were stained for 1 h with affinity-purified rabbit anti-RRM2B antibody followed by a 30-min exposure to anti-rabbit IgG-AlexaFluor 647, mouse anti-PYCR2 antibody followed by a 30-min exposure to anti-mouse IgG-AlexaFluor 594 and DAPI before mounted in ProLong® Gold Antifade Mountant (Thermo Fisher Scientific Inc.). Fluorescence images of RRM2B and PYCR2 were captured with wide-field DeltaVision deconvolution microscope (Applied Precision Inc., GE Healthcare Life Science, Pittsburgh PA.), equipped with 60x/1.42 N.A. oil-immersion objective lens. Both microscope and camera were controlled by SoftWorX application suite software. Stacks of optical section images, with image size of 512 × 512, were collected for all fluorochromes. All images were deconvolved by using SoftWorX software (Applied Precision Inc.), and later analyzed with VoloCITY software (PerkinElmer).

### Analysis of mitochondria morphology by Confocal Microscopy

HFF-hTERT cells expressing cox4-DsRed were transduced with pMSCVhyg vector expressing RRM2B or pLKO.1 lentiviral vectors expressing non-silencing shRNA and shRNAs directed to silence PYCR1, PYCR2 or both. Following hygromycin or puromycin selection, cells were seeded onto coverglasses in triplicate and fixed in 4% paraformaldehyde for 10 minutes at 37 °C. Fixed cells were washed with PBS and stained with DAPI before mounted in ProLong® Gold Antifade Mountant (Thermo Fisher Scientific Inc.). Mitochondrial morphology was visualized by Zeiss Upright LSM510 2-Photon microscope (Carl Zeiss, Oberkochen, Germany) and photographed. Mitochondrial morphology was categorized into three groups, mostly long tubular, intermediate phenotype and mostly fragmented. At least 100 cells were scored from each coverglass.

### Colony Formation Assay

Transduced HFF-hTERT cells following puromycin selection were seeded in 60 mm dishes at low density (200–500 cells per dish). Cell colonies were fixed and stained with 0.5% Crystal Violet in methanol:acetic acid (3:1, v/v) two to three weeks following seeding. For hydrogen peroxide sensitivity assays, cells were treated with hydrogen peroxide in serum free media for 10 minutes at 37 °C after overnight seeding. Treated cells were rinsed with complete media two times and incubated for 2-3 weeks before fixation and staining.

### Cell Proliferation Assay

Transduced HFF-hTERT cells were seeded in 6-well plates (1 × 10^5^ per well) following puromycin selection. Cell numbers were counted two days and three days after seeding. For hydrogen peroxide sensitivity assays, cells were treated with hydrogen peroxide in serum free media for 10 minutes at 37 °C after seeding for overnight. Treated cells were rinsed with complete media for two times and incubated for additional two days before cell counting.

### Cell Cycle Analysis

Transduced HFF-hTERT cells were harvested, stained in propidium iodide solution (0.05 mg/ml propidium iodide, 0.1% (w/v) sodium citrate and 0.1% (v/v) Triton X-100) and digested with RNAse A (2 μg/ml) before analysis by fluorescence-activated cell sorting.

### Zebrafish Maintenance

Zebrafish (*Danio rerio*) were obtained from zebrafish core facility of Taipei Medical University and maintained at 28 °C on a 14 h light/10 h dark cycle. All animal procedures were approved by Taipei Medical University Institutional Animal Care and Utilization Committee (TMU-IACUC). The methods were carried out in accordance with the approved guidelines. Embryos were incubated at 28 °C and different developmental stages were determined according to the Zebrafish Book^45^.

### Morpholino Knockdown of *pycr1, 2* and *3*

The antisense morpholino oligonucleotides (MOs), against zebrafish *pycr1*, *pycr2* and *pycr3*, were obtained from Gene Tools (Philomath, OR, USA). The *pycr1 and 2* MOs were designed according to a previous report^27^. The sequences of the *pycr* MOs were as follows: *pycr1* MO 5′-CAGCTCCGATAAATCCCACACTCA T-3′, *pycr2* MO 5′-CCG CTCCAATGAAGCCCACACTCAT-3′, and *pycr3* MO 5′-CTGAAGCACCTGACA GACTCATGGT-3′. Each MO was dissolved in 1 Danieau solution containing 0.5% phenol red to a concentration of 5 and 7.5 ng. The *pycr* MOs were injected into at the 1- to 2-cell stage of wild-type zebrafish embryos using a microinjection system consisting of a SZX16 stereomicroscope (Olympus, Tokyo, Japan) and an IM-300 Microinjector (Narishige, Scientific Instrument Lab., Tokyo, Japan). The MO-mediated effects were evaluated at 24 h and 48 h (2 dpf) after injection. The phenotypes of fish morphants were categorized in three groups, normal, mild and severe. The embryos morphants were observed under an Olympus IX70-FLA inverted fluorescence microscope (Olympus, Tokyo, Japan), photographed by the SPOT digital camera system (Diagnostic Instruments, Sterling Heights, Michigan, USA), and assembled by ImageJ program^46^.

### Statistical Analysis

We performed Student’s t tests with two-tailed distribution and two-sample equal variance in Excel (Microsoft, Redmond, WA, USA).

## Additional Information

**How to cite this article**: Kuo, M.-L. *et al.* PYCR1 and PYCR2 Interact and Collaborate with RRM2B to Protect Cells from Overt Oxidative Stress. *Sci. Rep.* **6**, 18846; doi: 10.1038/srep18846 (2016).

## Supplementary Material

Supplementary Information
