# Supplementary material for: PYCR1 and PYCR2 Interact and Collaborate with RRM2B to Protect Cells from Overt Oxidative Stress
Source: Sci Rep. 2016 Jan 6;6:18846. doi: 10.1038/srep18846 (PMC4702135; doi:10.1038/srep18846)

Supplementary Information

PYCR1 And PYCR2 Interact And Collaborate With RRM2B To Protect Cells From Overt Oxidative Stress

Mei-Ling Kuo[1], Mabel Bin-Er Lee[1,†], Michelle Tang[1,†], Willem den Besten[2], Shuya Hu[1], Michael J. Sweredoski[3], Sonja Hess[3], Chih-Ming Chou[4], Chun A. Changou[5,6,7], Mingming Su[8], Wei Jia[8], Leila Su[7] and Yun Yen[1,4,5,6,7*§]

[1] Department of Molecular Pharmacology, Beckman Research Institute at City of Hope, Duarte, CA 91010, USA

[2] Division of Biology and Biological Engineering, California Institute of Technology, Pasadena, CA 91125, USA

[3] Proteome Exploration Laboratory, Division of Biology and Biological Engineering, Beckman Institute, California Institute of Technology, Pasadena, CA, 91125

[4] Department of Biochemistry and Molecular Cell Biology, School of Medicine, College of Medicine, Taipei Medical University, Taipei, Taiwan 110

[5] Integrated Laboratory, Center of Translational Medicine, Taipei Medical University, Taipei, Taiwan 110

[6] Graduate Institute of Translational Medicine, College of Medical Science and Technology, Taipei Medical University, Taipei, Taiwan 110

[7] Graduate Institute of Cancer Biology and Drug Discovery, College of Medical Science and Technology, Taipei Medical University, Taipei, Taiwan 110

[8] University of Hawaii Cancer Center, HI 96813, USA

[†] These authors contributed to this work equally.

[*] Corresponding author

[§] Current address:

Yun Yen, M.D., Ph.D.

Graduate Institute of Cancer Biology and Drug Discovery

College of Medical Science and Technology

Taipei Medical University

No. 250, Wuxing Street, Xinyi District, Taipei, Taiwan 110.

y.yenmd@gmail.com

Methods

Quantitative Real Time Polymerase Chain Reaction (qRT-PCR)

The procedure was described previously with modifications[1]. Total RNA was isolated using TRIzol® reagent (Thermo Fisher Scientific Inc., Waltham, MA, USA), and cDNA was generated by SuperScript™ II Reverse Transcriptase (Thermo Fisher Scientific Inc.) according to the manufacturer's instructions. qRT-PCR was performed in the C1000 Thermal Cycler CFX96 Real-Time System (Bio-Rad Laboratories Inc., Hercules, CA,USA) in a 25 μl mix containing cDNA synthesized from 50 ng of total RNA, 0.4 μM of each primer and SYBR® Advantage® qPCR Premix (Clontech Laboratories Inc., Mountain View, CA, USA). Relative gene expression was calculated with the $\Delta\Delta$Ct method using β-ACTIN as an internal control. Primer sequences used for amplifications were as follows: PYCR1 forward: 5'- GGACAAGGTGAAGCTGGACT; PYCR1 reverse: 5'-AGGACGTGTCAATCCTTGC; PYCR2 forward: 5'-AGATGGGTGTGAACCTGACA and PYCR2 reverse: 5'-CTTCACAGCCAGAAACAGGA. β-ACTIN primer sequences were described previously[1].

Mass Spectrometric and Data Analyses

All experiments were performed on a nanoflow LC system, EASY-nLC 1000 coupled to a hybrid linear ion trap Orbitrap Classic mass spectrometer (Thermo Fisher Scientific Inc., Bremen, Germany) equipped with a nanoelectrospray ion source (Thermo Fisher Scientific Inc.). Solvent A was 97.8% H_2O, 2% ACN, and 0.2% formic acid and solvent B was 19.8% H_2O, 80% ACN, and 0.2% formic acid. For the LC-MS/MS experiments, 1 μg of digested peptides were directly loaded at a flow rate of 500 nL/min onto a 16-cm

analytical HPLC column (75 μm ID) packed in-house with ReproSil-Pur C_{18}AQ 3 μm resin (120 Å pore size, Dr. Maisch, Ammerbuch, Germany). The column was heated at 30°C using a column heater. After loading the peptides were separated with a 119 min gradient at a flow rate of 350 nL/min using the following gradient: 14-30% Solvent B (110 min), 30-100% B (1 min), and 100% B (8 min). The Orbitrap was operated in data-dependent acquisition mode to automatically alternate between a full scan (*m*/*z*=300–1700) in the Orbitrap and subsequent 15 CID MS/MS scans in the linear ion trap. CID was performed with helium as collision gas at a normalized collision energy of 35% and 30 ms of activation time. For the database search, mgf files were generated from the raw data using ReadWforMascot. The mgf files were searched against the human IPI database (v3.54) with a decoy using Mascot MS/MS ion search (Matrix Science Inc, Boston, MA, USA). Typical search parameters: trypsin with a maximum of two missed cleavages, carbamidomethylation of Cys as fixed modification, and methionine oxidation and N-acetylation as variable modifications. For the mass accuracy a window of ± 10 ppm was set for the precursor ion and ± 0.5 Da for the MS/MS fragment ion mass values. Subsequently, the Mascot files were imported in the Scaffold 3.5.1. software with a minimum of 2 peptides per protein and minimum identification probabilities for both protein and peptide set at 90% resulting in a false discovery rate of less than 1%.

Targeted Metabolite Analysis

HFF-hTERT cells were transduced with pSUPER.retro.puro retroviruses expressing shNS, shPYCR1/2-A, shPYCR1/2-B and shRRM2B and selected with puromycin. Ten million cells from each cell line were harvested one week after infection. Frozen cell pellets were thawed on an ice-bath. Appropriate weight of homogenizer beads and 150 μl

of cold water were added to the sample for first-step homogenization. An aliquot of 750 µl methanol and chloroform mixture (2:1 = v/v) was added to the sample for second-step homogenization and extraction. The extracts were centrifuged at 4 °C and 14, 500 rpm for 20 min and 20 µl of the supernatant was added to a 96-well Biocrates Kit plate (Biocrates Life Sciences, Innsbruck, Austria) for amino acid quantitation. Each 10 µl of amino acid standards and stable-isotope labeled internal standards were also added to the kit plate to establish a quantitation curve. Fifty µl of freshly prepared 5% phenylisothiocyanate (PITC) solution (1 vol. of PITC and 19 vol. of ethanol: water: pyridine (1:1:1, v/v/v)) was added to each well plate for amino acid derivatization. After samples were dried under nitrogen, 300 µl of extraction solvent (5 mM ammonium acetate in methanol) was added to each sample and the kit plate was gently shaken at room temperature for 30 min. The sample extracts were filtered through the 0.45 µm membrane and each aliquot of 50 µl of sample was further diluted with 40% methanol before LC-MS/MS analysis. Acquity ultra performance liquid chromatography coupled with Xevo TQ-S mass spectrometer (UPLC-MS/MS, Waters Corp., Milford, MA, USA) was used for targeted metabolite analysis. The mobile phases included (A) water containing 0.2% formic acid, and (B) acetonitrile containing 0.2% formic acid. Each 5 µL of sample was separated on a BEH C18 1.7 µm 2.1 x 75 mm column (Waters, Milford, MA, USA) at a temperature of 50 °C and at a flow rate of 900 µl/min. The following elution program was used for separation; 0-0.5min (1% B), 3 min (15% B), 5.4 min (70% B), 5.6-6.1 min (100% B), and 6.1-6.6 min (1% B).

Supplementary Table S1. PCR primer sequences

cDNA	Primer sequence
RRM2B	Forward: 5’ AAAGGATCCGCCACCATGGGCGACCCGGAAAGGCC
	Reverse: 5’ AAAACTCGAGTTAAAAATCTGCATCCAAGGTGAAGACG
Flag-RRM2B	Forward: 5’ AAAGGATCCGCCACCATGGATTACAAGGATGACGACGATAAGATGGGCGACCCGGAAAGGCC
	Reverse: 5’ AAAACTCGAGTTAAAAATCTGCATCCAAGGTGAAGACG
RRM2B-Flag	Forward: 5’ AAAGGATCCGCCACCATGGGCGACCCGGAAAGGCC
	Reverse: 5’ AAAACTCGAGTTACTTATCGTCGTCATCCTTGTAATCAAAATCTGCATCCAAGGTGAAGACG
HA-RRM2B	Forward: 5’ AAAGGATCCGCCACCATGGGCTACCCCTACGACGTGCCCGACTACGCCATGGGCGACCCGGAAAGGCC
	Reverse: 5’ AAAACTCGAGTTAAAAATCTGCATCCAAGGTGAAGACG
RRM2B-HA	Forward: 5’ AAAGGATCCGCCACCATGGGCGACCCGGAAAGGCC
	Reverse: 5’ AAAACTCGAGTTAGGCGTAGTCGGGCACGTCGTAGGGGTAAA

	AATCTGCATCCAAGGTGAAGACG
OneStrep-RRM2B (1st step)	Forward: 5' GGTGGAGGTTCTGGCGGTGGATCGGGAGGTTCAGCGTGGAGCCACCCGCAGTTCGAAAAAATGGGCGACCCGGAAAGGCC
	Reverse: 5' AAAACTCGAGTTAAAAATCTGCATCCAAGGTGAAGACG
OneStrep-RRM2B (2nd step)	Forward: 5' AAAGGATCCGCCACCATGGGCAGCGCATGGAGTCATCCTCAATTCGAGAAAGGTGGAGGTTCTGGCGGTG
	Reverse: 5' AAAACTCGAGTTAAAAATCTGCATCCAAGGTGAAGACG
RRM2B-OneStrep (1st step)	Forward: 5' AAAGGATCCGCCACCATGGGCGACCCGGAAAGGCC
	Reverse: 5' CCGATCCACCGCCAGAACCTCCACCTTTCTCGAATTGAGGATGACTCCATGCGCTAAAATCTGCATCCAAGGTGAAGACG
RRM2B-OneStrep (2nd step)	Forward: 5' AAAGGATCCGCCACCATGGGCGACCCGGAAAGGCC
	Reverse: 5' AAAACTCGAGTTATTTTTCGAACTGCGGGTGGCTCCACGCTGAACCTCCCGATCCACCGCCAGAAC
HA-PYCR1	Forward: 5' AAAGGATCCGCCACCATGGGCTACCCCTACGACGTGCCCGACTACGCCATGAGCGTGGGCTTCATCGG
	Reverse: 5' AAAACTCGAGTCAATCCTTGCCCGCTGGGG

HA-PYCR2	Forward: 5' AAAGGATCCGCCACCATGGGCTACCCCTACGACGTGCCCGACTACGCCATGAGCGTGGGCTTCATCGG
	Reverse: 5' AAAACTCGAGTTAGTCCTTCTTGCCTCCCAG
HA-PYCRL	Forward: 5' AAAGGATCCGCCACCATGGGCTACCCCTACGACGTGCCCGACTACGCCATGGCAGCTGCGGAGCCGTC
	Reverse: 5' AAAACTCGAGCTACTTTCTGCTGAGCTCCTTG

Supplementary Table S2. Target sequences of shRNA vectors

Vector	Renamed as	TRC Clone ID	Target sequence (sense)
pSUPER.retro.puro-PYCR1-A			GCCCACAAGATAATGGCTA
pSUPER.retro.puro-PYCR1-B	pSUPER.retro.puro - PYCR1/2-A		ACAAGGAGACGGTGCAGCA
pSUPER.retro.puro-PYCR1-C			GCGCCGACATTGAGGACAG
pSUPER.retro.puro-PYCR2-A	pSUPER.retro.puro - PYCR1/2-B		GCAACAAGGAGACGGTGAA
pSUPER.retro.puro-PYCR2-B			CCATCAGCTCTGTGGAGAA
pSUPER.retro.puro-PYCR2-C			CCAAGATGCTGCTGGACTC
pLKO.1/TRC-PYCR1-A	pLKO.1/TRC-PYCR1/2-A	TRCN0000038979	CACAGTTTCTGCTCTCAGGAA
pLKO.1/TRC-PYCR1-B		TRCN0000038980	CCCTTCATCCTGGATGAAAT
pLKO.1/TRC-PYCR1-C		TRCN0000038981	GAGGGTCTTCACCCACTCCTA
pLKO.1/TRC-PYCR1-D		TRCN0000038982	CCTGCTCATCAACGCTGTGGA
pLKO.1/TRC- PYCR1-E		TRCN0000038983	TGAGAAGAAGCTGTCAGCGTT
pLKO.1/TRC-PYCR2-A		TRCN0000046368	GCCCTTAAGAAGACCCTCTTA
pLKO.1/TRC- PYCR2-B		TRCN0000046369	CTGTCGGCTCACAAGATAATA
pLKO.1/TRC- PYCR2-C		TRCN0000046370	CGTCCTGTTTCTGGCTGTGAA
pLKO.1/TRC-PYCR2-D		TRCN0000046371	CCATGCCAGCTTAAGGACAAT
pLKO.1/TRC- PYCR2-E		TRCN0000046372	GACCCTCTTAGACAGAGTGAA

Supplementary Figure S1. Induction of epitope-tagged RRM2B proteins by doxycycline in 293 T-REx cells. Stable 293 T-REx cell lines engineered to express empty vector, untagged or epitope-tagged RRM2B were treated (+) with 1 μg/ml doxycycline (DOX) for 24 hours or left untreated (-) as controls. Denatured whole cell lysates separated on gels via electrophoresis and were transferred to membranes and blotted with antibodies to RRM2B, Flag, HA and RRM1. GAPDH was used as control to ensure equal loading of each lane. N-: N-terminally tagged with an epitope. C-: C-terminally tagged with an epitope.

Supplementary Figure S2. Interaction of HA-tagged PYCRL and Flag-tagged RRM2B. 293T cells were co-transfected with either RRM2B or Flag-RRM2B vectors with HA-PYCRL vector and lysed 48 hours later. Whole cell lysates were subjected to immunoprecipitation with antibodies to HA or Flag. Denatured whole cell lysates (WCL) and immune complexes electrophoretically separated on gels were transferred to membranes and blotted with antibodies to HA, Flag and RRM2B.

Supplementary Figure S3. Predicted PYCR1-RRM2B complex. The NAD-bound human PYCR1 (PDB ID 2IZZ, R = 1.95) pentamer and the human RRM2B (PDB ID 3HF1, R = 2.60) dimer structures were downloaded and used in docking study. Protein-protein docking was performed using Hex 8.0.0 and the best predicted model was presented, where RRM2B fills in to contact PYCR1 pentamer chains BCDE through their inter-chain binding domains. Protein structures were shown in ribbon renderings, where PYCR1 chain A was colored in cyan, chain B in yellow, chain C in purple, chain D in green and chain E in pink. NAD was in ball-and-stick and color-coded as atom types (carbon in white, oxygen in red and nitrogen in blue). RRM2B chain A was colored in

light green and chain B in light pink. Iron ions were rendered in space and colored in red. Surface representation of the receptor, PYCR1, was shown in white and the ligand, RRM2B, was shown in yellow. SYBYLx2.1.1 (Tripos-Certara) was used in the structural analysis and presentation.

Supplementary Figure S4. Silencing of PYCR1 and PYCR2 by shRNA vectors. HFF-hTERT cells were transduced with (A) pSUPER.retro.puro retroviruses or (B) pLKO.1/TRC lentiviruses expressing shRNAs that were designed to silence either PYCR1 or PYCR2. Transduced cells were selected with puromycin for at least two days. Whole cell lysates were subjected to Western blot analysis to examine efficiency of silencing. β-ACTIN was used as control to ensure equal loading of each lane. pSUPER.retro.puro-shPYCR1-B (renamed as pSUPER.retro.puro-shPYCR1/2-A) and -shPYCR2-A (renamed as pSUPER.retro.puro-shPYCR1/2-B), pLKO.1-shPYCR1-A (renamed as pLKO.1-shPYCR1/2-A), -C, and -E as well as pLKO.1-shPYCR2-A and -D silenced both PYCR1 an PYCR2. pLKO.1-shPYCR1-D specifically silenced PYCR1 and pLKO.1-shPYCR2-B and -D specifically silenced PYCR2. (C) Total RNAs from HFF-hTERT cells transduced with pSUPER.retro-puro-shNS, -shPYCR1/2-A and shPYCR1/2-B retroviruses were isolated. Real-time RT-PCR was performed to quantify expression levels of PYCR1 and PYCR2. Data were plotted as % of shNS control. (D) Alignment of PYCR1 and PYCR2 sequences within the shRNA targeting regions.

Supplementary Figure S5. Morpholino (MO) knockdown of *pycr* in zebrafish affects somatogenesis and cerebral hemorrhage during embryonic stages. (A) Each *pycr* MO (7.5 ng) was injected into wild-type zebrafish embryos at the 1- to 2-cell stage and evaluated at 48 hours post fertilization (hpf). The major defects of *pycr1*-morphants (left

panel) or *pycr3*-morphants (right panel) were reduced body length and a curly tail. The *pycr2*-MO injected embryos exhibited no obvious abnormality, but the morphants shows cerebral hemorrhage (middle panel). Arrows indicate area of hemorrhage. Lateral views of representative wild-type control (Ctrl) or MO-injected fish were photographed at 48 hpf. Bar: 100 μm. (B) Phenotypic distributions of *pycr1, pycr2* and *pycr3* morphants.

Supplementary Figure S6. Morpholino (MO) knockdown of two *pycr* genes in zebrafish caused severe defects during embryonic development. (A) Two *pycr* MOs (5 or 7.5 ng of each MO) were co-injected into wild-type zebrafish embryos at the 1- to 2-cell stage. Lateral views of representative wild-type control (Ctrl) or embryos co-injected with *pycr1* and *pycr2* MOs, *pycr1* and *pycr3* MOs or *pycr2* and *pycr3* MOs were photographed at 24 hours post fertilization (hpf), 24 hpf and 48 hpf respectively. (left panel: low magnification; right panel: high magnification) Co-injection with two *pycr* MOs resulted in reduced body length, a curly tail or abnormal somite phenotypes at 48 hpf. The control embryo exhibited chevron-shaped somites, but flatter morphology and shorter somites were observed in *pycr* MOs-injected embryos. The dash-line depicts the shape of somites. Scale bars: 100 μm. (B) Phenotypic distributions for embryos co-injected with two *pycr* MOs. For all co-injections, we observed a marked increase in the percentage of affected embryos with severe phenotypes.

Supplementary Figure S7. Alignment of RRM2B isoforms and mitochondria targeting sequence in isoform 2. Predicted peptide sequences of RRM2B isoform 1, 2 and 3 were aligned by Constraint-based Multiple Alignment Tool (COBALT). All of the three sequences were analyzed by MitoProt to predict mitochondria targeting sequence (MTS). A predicted MTS was identified at the N-terminus of isoform2 (underlined in

red).

Supplementary Figure S8. Targeted metabolite analysis in HFF-TERT cells expressing shNS, shPYCR1/2 and shRRM2B. HFF-hTERT cells were transduced with pSUPER.retro.puro retroviruses expressing shNS, shPYCR1/2-A, shPYCR1/2-B and shRRM2B. Infected cells were selected with puromycin and harvested for amino acids quantification analysis. Data were plotted as % of shNS control.

REFERENCES

1. Kuo, M.L., *et al.* RRM2B suppresses activation of the oxidative stress pathway and is up-regulated by p53 during senescence. *Sci. Rep.* **2**, 822 (2012).

Supplementary Figure S1

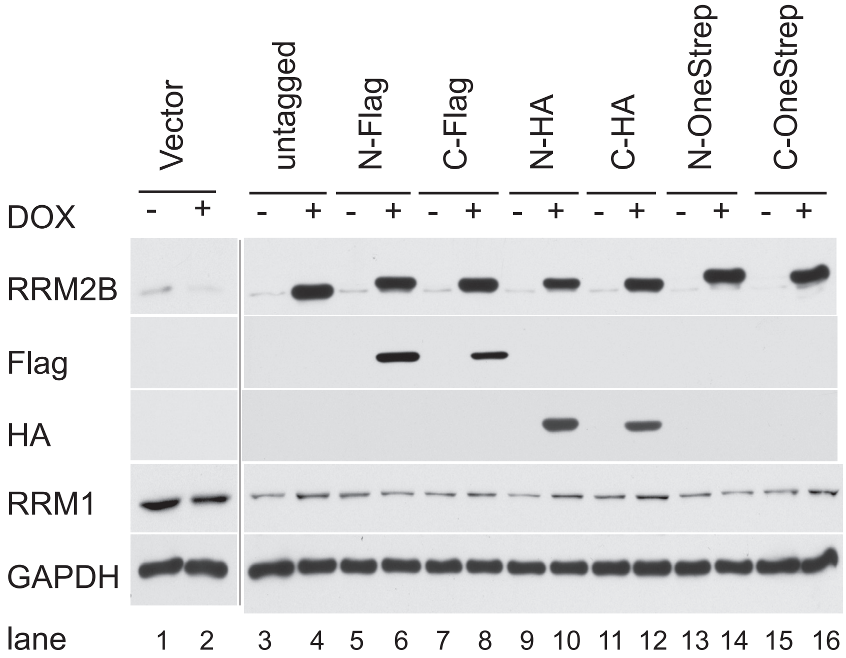

Supplementary Figure S2

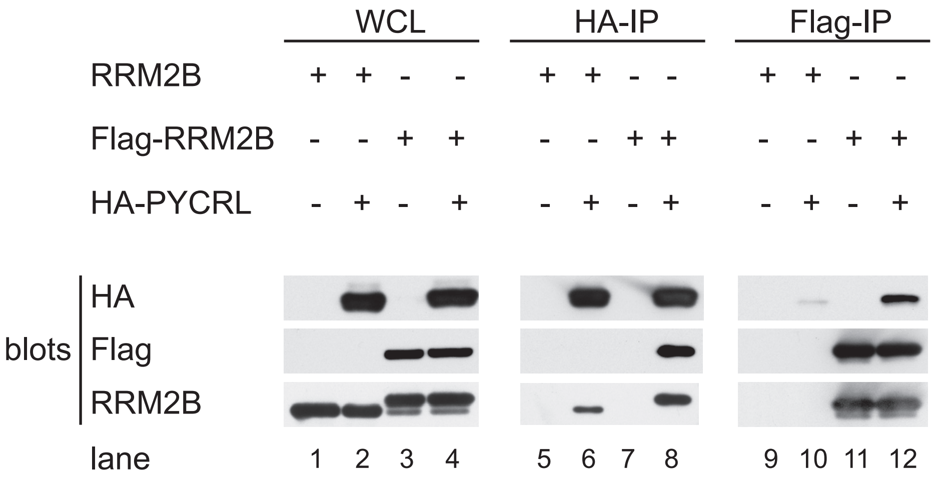

Supplementary Figure S3

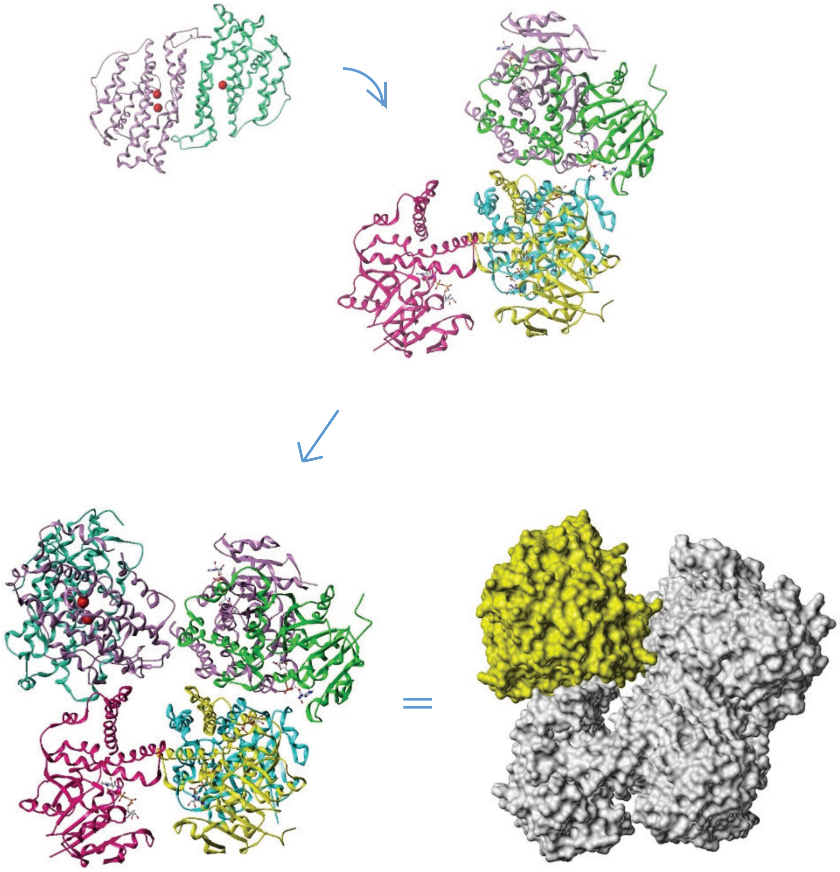

Supplementary Figure S4

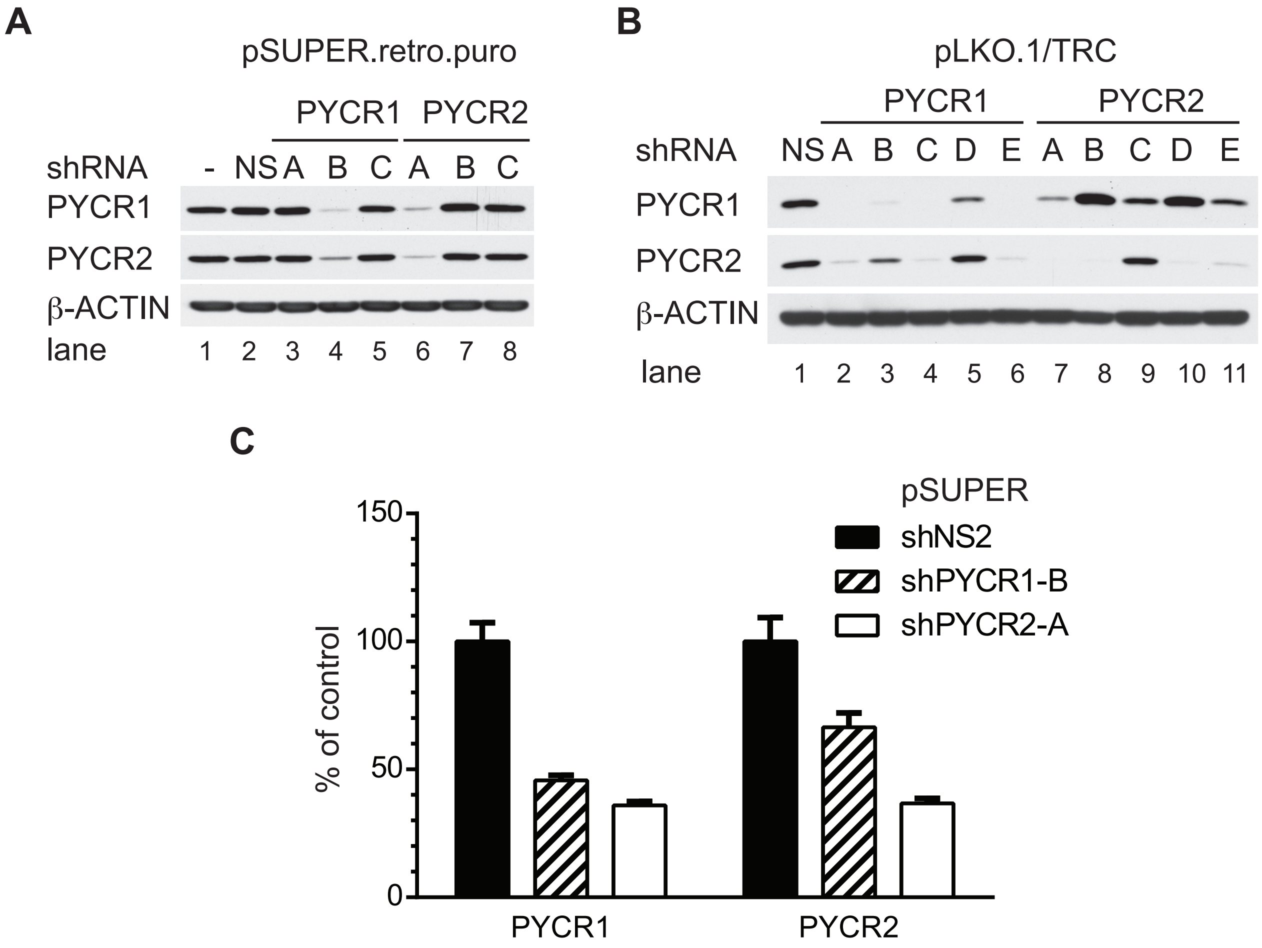

D

pSUPER.retro.puro
shPYCR1-B
PYCR1: ACAAGGAGACGGTGCAGCA
PYCR2: ACAAGGAGACGGTG***A***AGCA

shPYCR2-A
PYCR2: GCAACAAGGAGACGGTGAA
PYCR1: ***A***CAACAAGGAGACGGTG***C***A

pLKO.1/TRC
shPYCR1-A
PYCR1: CACAGTTTCTGCTCTCAGGAA
PYCR2: CAC***G***GT***G***TC***C***GC***G***CTCAGGAA

Supplementary Figure S5

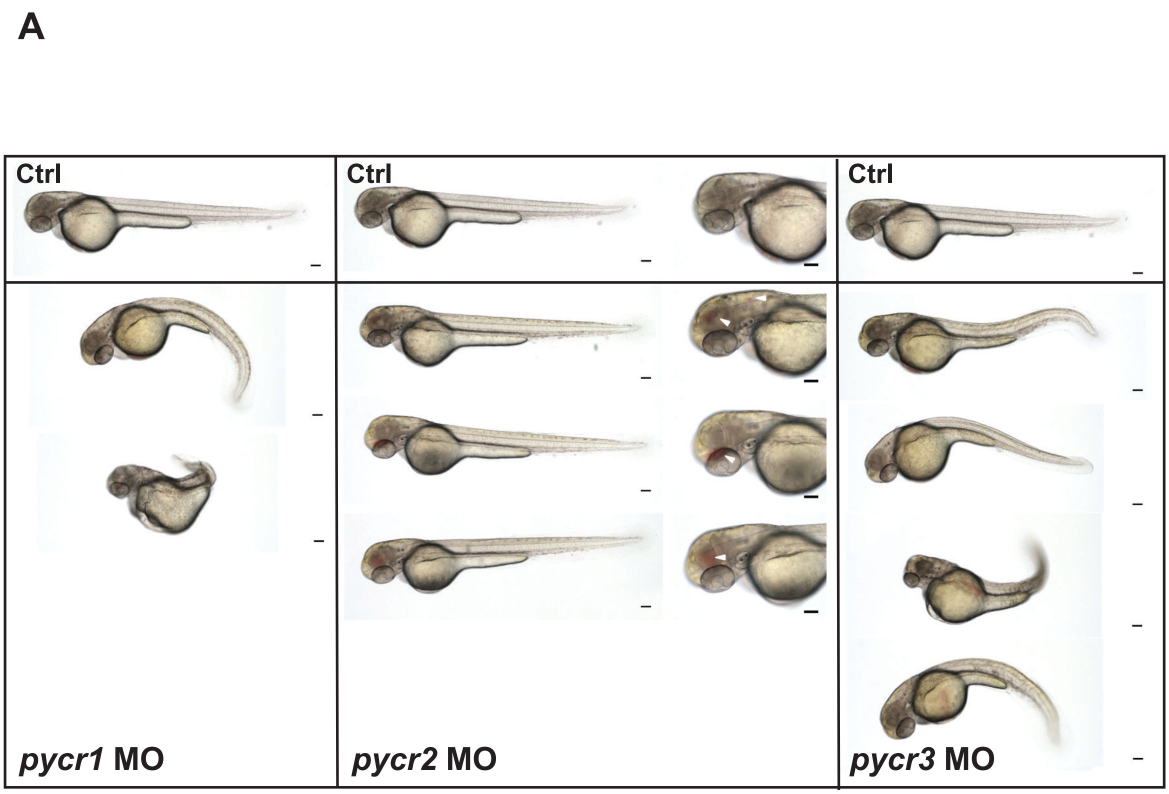

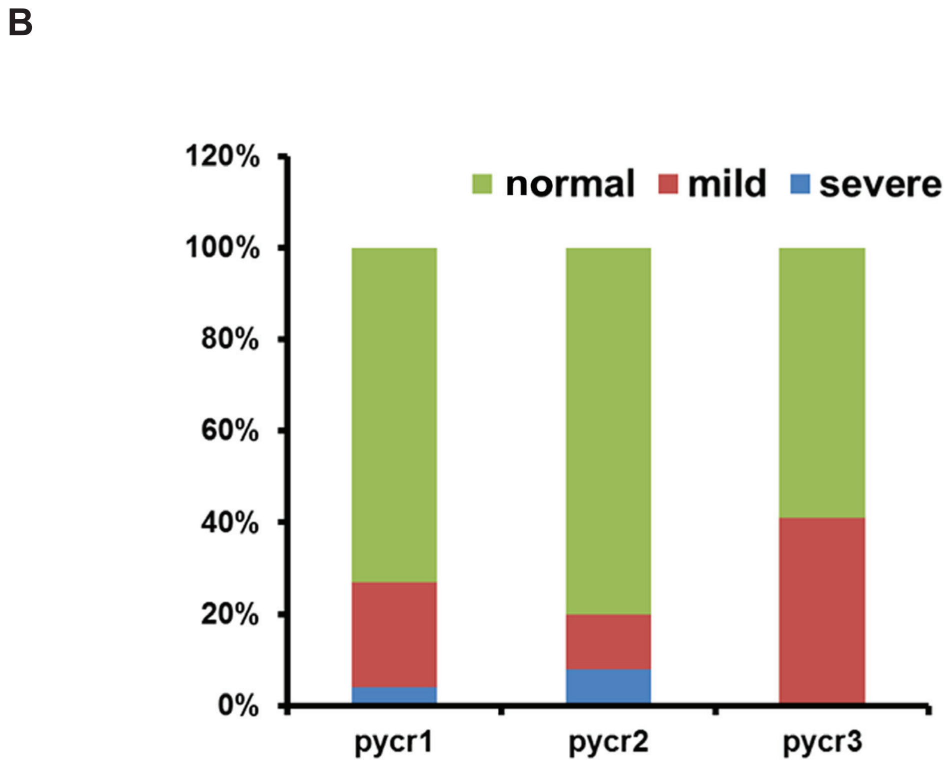

7.5ng / injection	Pycr1 MO (n=26)	Pycr2 MO (n=26)	Pycr3 MO (n=29)
severe	1 (4%)	2 (8%)	0 (0%)
mild	6 (23%)	3(12%)	12 (41%)
normal	19 (73%)	21(80%)	17 (59%)

Supplementary Figure S6

A

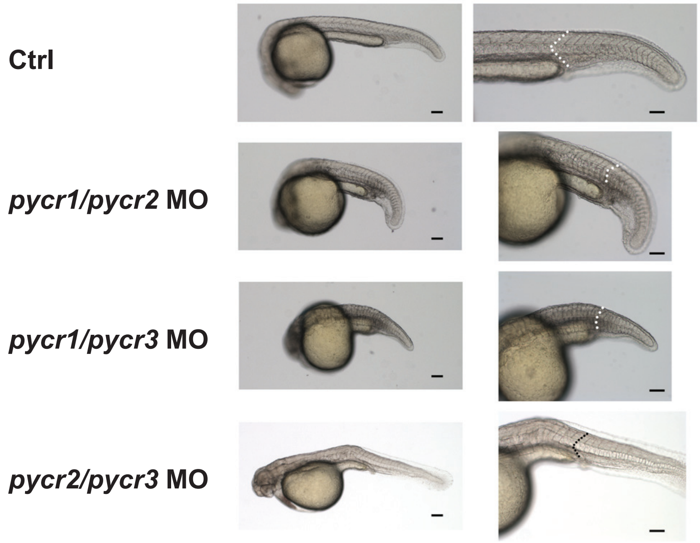

B

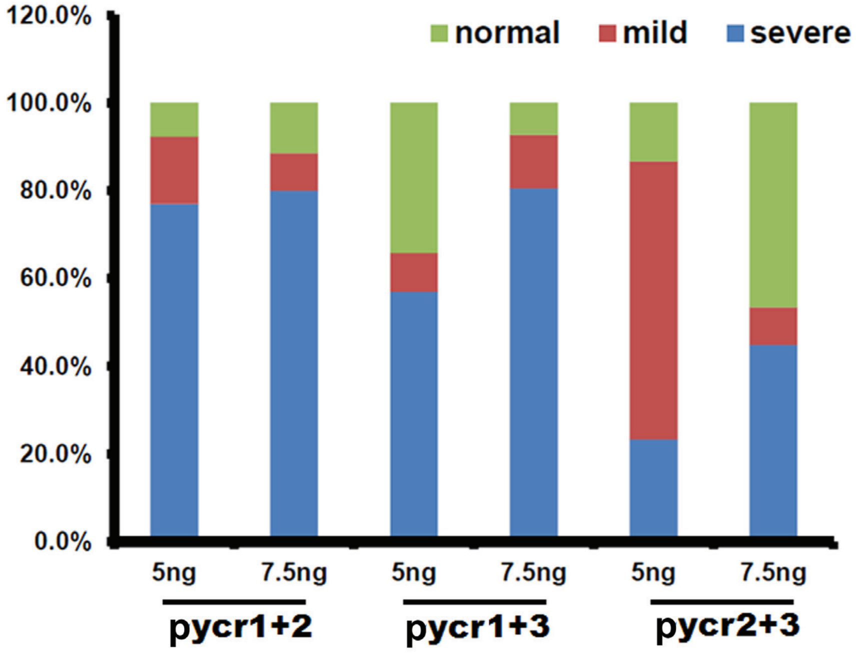

	pycr MO 1+2		pycr MO 1+3		pycr MO 2+3	
	5ng (n=39)	7.5ng (n=74)	5ng (n=79)	7.5ng (n=73)	5ng (n=30)	7.5ng (n=58)
severe	30 (76.9%)	63 (80.0%)	45 (57.0%)	59 (80.5%)	7 (23.3%)	26 (44.8%)
mild	6 (15.4%)	4 (8.6%)	7 (8.9%)	10 (12.2%)	19 (63.3%)	5 (8.6%)
normal	3 (7.7%)	7 (11.4%)	27 (34.2%)	4 (7.3%)	4 (13.3%)	27 (46.6%)

Supplementary Figure S7

```
isoform 1  1    ----------------------------------------------------------------MG-DPERPEAAGLDQD  15
isoform 2  1    MLLLRLPPHRSHASPLDCKLQDRCRKCYSPRSGQACPPALAAAWLRRCERRGGRPRGGRRKELTLGLRPARCSAPGPAKD  80
isoform 3  1    ----------------------------------------------------------------MG-DPERPEAAGLDQ-  14

isoform 1  16   E-------RSSSDTNESEIKSNEEPLLRKSSRRFVIFPIQYPDIWKMYKQAQASFWTAEEVDLSKDLPHWNKLKADEKYF  88
isoform 2  81   DAWRPQAGRSSSDTNESEIKSNEEPLLRKSSRRFVIFPIQYPDIWKMYKQAQASFWTAEEVDLSKDLPHWNKLKADEKYF  160
isoform 3  15   ----------------------------------------------------------DEVDLSKDLPHWNKLKADEKYF  36

isoform 1  89   ISHILAFFAASDGIVNENLVERFSQEVQVPEARCFYGFQILIENVHSEMYSLLIDTYIRDPKKREFLFNAIETMPYVKKK  168
isoform 2  161  ISHILAFFAASDGIVNENLVERFSQEVQVPEARCFYGFQILIENVHSEMYSLLIDTYIRDPKKREFLFNAIETMPYVKKK  240
isoform 3  37   ISHILAFFAASDGIVNENLVERFSQEVQVPEARCFYGFQILIENVHSEMYSLLIDTYIRDPKKREFLFNAIETMPYVKKK  116

isoform 1  169  ADWALRWIADRKSTFGERVVAFAAVEGVFFSGSFAAIFWLKKRGLMPGLTFSNELISRDEGLHCDFACLMFQYLVNKPSE  248
isoform 2  241  ADWALRWIADRKSTFGERVVAFAAVEGVFFSGSFAAIFWLKKRGLMPGLTFSNELISRDEGLHCDFACLMFQYLVNKPSE  320
isoform 3  117  ADWALRWIADRKSTFGERVVAFAAVEGVFFSGSFAAIFWLKKRGLMPGLTFSNELISRDEGLHCDFACLMFQYLVNKPSE  196

isoform 1  249  ERVREIIVDAVKIEQEFLTEALPVGLIGMNCILMKQYIEFVADRLLVELGFSKVFQAENPFDFMENISLEGKTNFFEKRV  328
isoform 2  321  ERVREIIVDAVKIEQEFLTEALPVGLIGMNCILMKQYIEFVADRLLVELGFSKVFQAENPFDFMENISLEGKTNFFEKRV  400
isoform 3  197  ERVREIIVDAVKIEQEFLTEALPVGLIGMNCILMKQYIEFVADRLLVELGFSKVFQAENPFDFMENISLEGKTNFFEKRV  276

isoform 1  329  SEYQRFAVMAETTDNVFTLDADF  351
isoform 2  401  SEYQRFAVMAETTDNVFTLDADF  423
isoform 3  277  SEYQRFAVMAETTDNVFTLDADF  299
```

Supplementary Figure S8

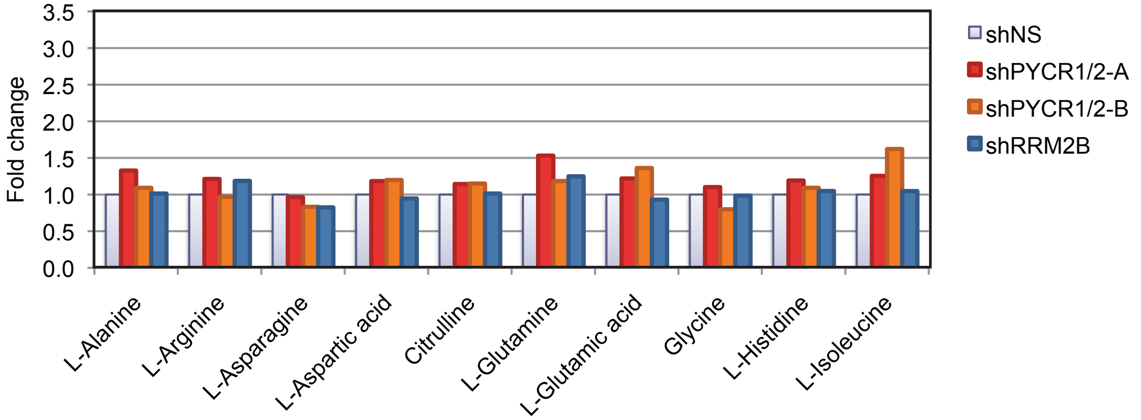

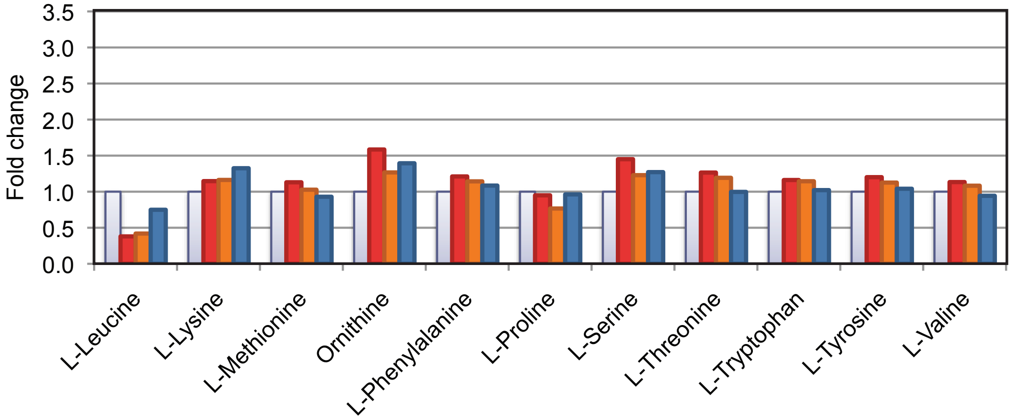

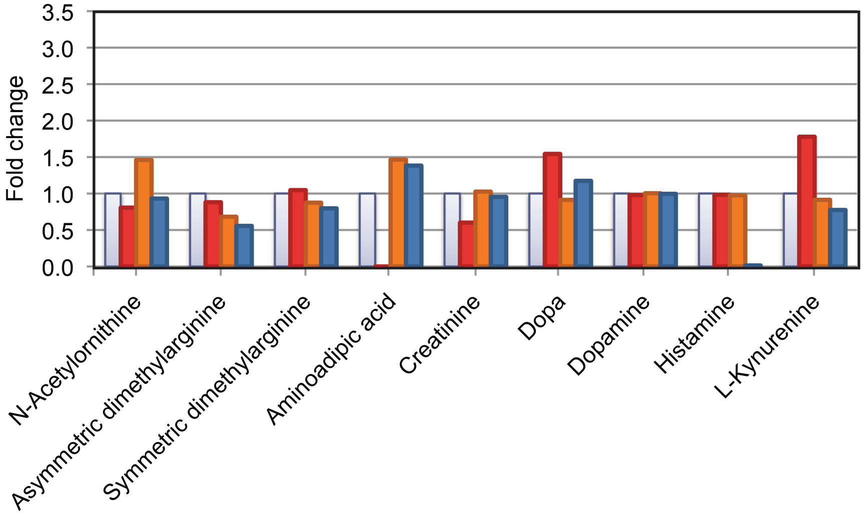

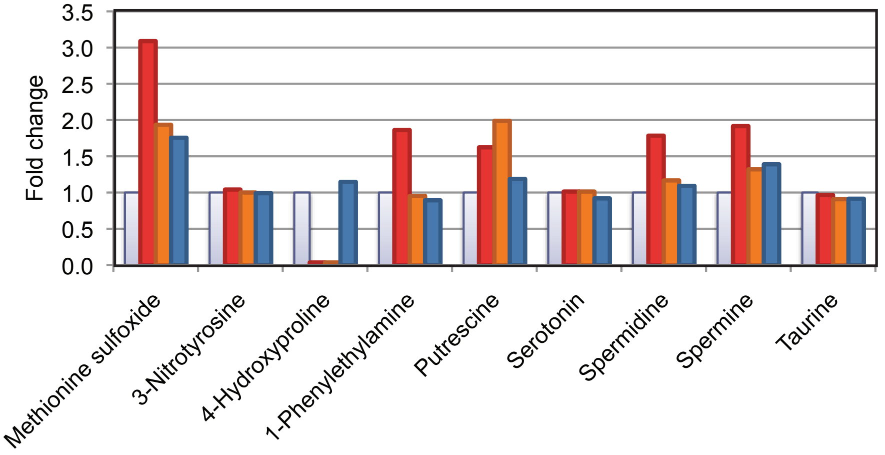

Supplement: Supplementary Information [file srep18846-s1.pdf]
